# Sparse System Identification of Leptin Dynamics in Women With Obesity

**DOI:** 10.3389/fendo.2022.769951

**Published:** 2022-04-05

**Authors:** Md. Rafiul Amin, Divesh Deepak Pednekar, Hamid Fekri Azgomi, Herman van Wietmarschen, Kirstin Aschbacher, Rose T. Faghih

**Affiliations:** ^1^ Department of Electrical and Computer Engineering, University of Houston, Houston, TX, United States; ^2^ Department of Nutrition and Health, Louis Bolk Instituut, Bunnik, Netherlands; ^3^ Department of Psychiatry, Weill Institute for Neurosciences, University of California, San Francisco, San Francisco, CA, United States

**Keywords:** obesity, leptin, cortisol, state-space, sparse recovery, deconvolution, correlation, grange-causality

## Abstract

The prevalence of obesity is increasing around the world at an alarming rate. The interplay of the hormone leptin with the hypothalamus-pituitary-adrenal axis plays an important role in regulating energy balance, thereby contributing to obesity. This study presents a mathematical model, which describes hormonal behavior leading to an energy abnormal equilibrium that contributes to obesity. To this end, we analyze the behavior of two neuroendocrine hormones, leptin and cortisol, in a cohort of women with obesity, with simplified minimal state-space modeling. Using a system theoretic approach, coordinate descent method, and sparse recovery, we deconvolved the serum leptin-cortisol levels. Accordingly, we estimate the secretion patterns, timings, amplitudes, number of underlying pulses, infusion, and clearance rates of hormones in eighteen premenopausal women with obesity. Our results show that minimal state-space model was able to successfully capture the leptin and cortisol sparse dynamics with the multiple correlation coefficients greater than 0.83 and 0.87, respectively. Furthermore, the Granger causality test demonstrated a negative prospective predictive relationship between leptin and cortisol, 14 of 18 women. These results indicate that increases in cortisol are prospectively associated with reductions in leptin and vice versa, suggesting a bidirectional negative inhibitory relationship. As dysregulation of leptin may result in an abnormality in satiety and thereby associated to obesity, the investigation of leptin-cortisol sparse dynamics may offer a better diagnostic methodology to improve better treatments plans for individuals with obesity.

## 1 Introduction

Obesity, dubbed the “Global Epidemic” by the World Health Organization, is said to cause or aggravate various other health problems, worsening one’s life expectancy (1, 2). The prevalence of obesity is increasing worldwide at an alarming rate. The estimate shows that about 38% of the adult American population suffer from obesity (3). Obesity is associated with a reduced life expectancy (4). It also increases the risk of other chronic disorders such as diabetes and cardiovascular disease (5). Available studies suggest different lifestyle interventions for treating obesity such as a low-calorie diet, increased physical activity, and bariatric surgery (6). Nevertheless, many studies have pointed the determinants of failure of such approaches including adherence to lifestyle interventions (7), genetic background, adaptive changes in basal metabolic rate, hunger and satiety hormones that occur with weight loss (8). While there exists pathogenic approaches to treat the condition through studying its development, a salutogenic model focused on holistic wellbeing might be more effective as both a preventive and remedial measure (9, 10). For example, obesity can be conceptualized as a deficiency of energy regulation - i.e., the brain may fail to respond to negative feedback hormonal signals from adipose tissue, thereby perpetuating non-homeostatic eating behaviors that drive obesity. Thus, identifying the role of neuroendocrine hormones and adipokines in facilitating brain-adipose communication and energy regulation may inform novel strategies for the treatment and prevention of obesity. In this research, we propose models to understand leptin and cortisol behavior, which, in the future, can further be generalized for other relevant hormones, such as growth hormone, insulin, and ghrelin. Leptin is a signaling hormone and adipokine that is essential to activating central nervous system (CNS) networks involved in the suppression of appetite. It regulates food intake, metabolism, energy expenditure, and body weight (11). Although yet to be proven, Jacquier et al. (12) mathematically demonstrated how leptin resistance can be a result of persistent leptin infusion. They further pointed out that temporal alteration in some parameter values related to food intake can shift the equilibrium. The shift in equilibrium can lead to leptin resistance and obesity state. Blood leptin levels also correlate with changes in fat mass (13). Leptin is a hormone produced and secreted primarily by adipocytes. Hence, excessive amounts of adipose tissue in the body correspond to an increase in leptin production rate, and thus, higher serum leptin levels (11). Leptin interacts with the hypothalamus-pituitary-adrenal-axis (HPA-axis), thereby constituting an important biological mechanism whereby experiences of psychological stress may influence or be influenced by body weight and energy regulation (14, 15).

Another frequently studied hormone in obesity research is the glucocorticoid hormone cortisol (16). In response to different physiological demands such as blood glucose regulation, inflammation suppression, physiological stress, significant HPA-axis responses are induced across all ages and genders (17). Björntorp et al. (16) compared several studies and concluded that the association between obesity and cortisol is complicated. They concluded that there are several factors such regulatory systems, including the HPA, gonadal, growth-hormone, leptin axes, the sympathetic nervous system, the central adrenergic, serotoninergic, and dopaminergic systems that are related to obesity. Despite cortisol’s short half-life and circadian secretion pattern, most studies use cortisol measures such as serum, urine or salivary cortisol that do not reflect long-term cortisol exposure (18). Björntorp et al. (16) suggest that it is necessary to examine the cortisol secretion process to further understand the roots of obesity.

In an attempt to study obesity, various models based on ordinary differential equations considering regulations in energy and metabolism (19–23), as well as on the effects of different hormones such as ghrelin, cholecystokinin, and leptin have been hypothesized. Traditional studies perform statistical analysis on the measured serum hormone levels (24, 25). Some models include leptin, leptin resistance, and leptin receptors to understand obesity (12, 26, 27). Other studies show the association of glucocorticoids and weight gain (28, 29). Since obesity is associated with the polypeptide hormone, leptin (11, 12, 14), and the glucocorticoid hormone, cortisol (16, 30), studying them will prove vital in designing an approach to treating the condition. For example, we cannot measure leptin resistance in a living human, nevertheless, investigation of the association between cortisol and leptin through mathematical modeling may lead to some insights into the presence of leptin resistance.

Aschbacher et al. (23) propose a mathematical model based on the systems-level understanding of the HPA-leptin axis. Incorporating leptin in the model helped them to obtain three parameters: (I) inhibitory cortisol feedback signal, (II) Adrenocorticotropic hormone (ACTH)-adrenal interaction, and (III) leptin-cortisol proportionality. The authors suggested that the leptin-cortisol relationship may be important for understanding the neuroendocrine starvation response, and that poor system control may ultimately contribute to obesity. The mathematical model presented in the research by Aschbacher et al. (23) explains the rate of changes in cortisol concentration, ACTH, and leptin dynamics. The model used in this paper is an extension of the HPA system dynamics model used previously in (31) incorporating leptin’s impact on cortisol. Though leptin regulation is dependent on its relation with different hormones, understanding its behavior requires the study of leptin secretion and regulation. Investigating cortisol and leptin separately, and based on their physiology, will be a vital approach in interpreting how they behave concerning each other and other systems of the body. Moreover, cortisol and leptin each have circadian and ultradian rhythms, which our approach may better characterize. Jacquier et al. (12) introduce a mathematical model to study leptin resistance based on leptin synthesis and receptor activity. They study the changes in plasma leptin concentration, the density of leptin receptors, and food intake by varying leptin infusion and food consumption. This model studies leptin resistance dynamics based on many parameters such as food intake, fat mass, leptin receptors, and leptin. However, it is challenging to simultaneously collect this kind of clinical data. Therefore, we propose a simplified model considering only plasma leptin levels as the observation. As leptin secretion is a pulsatile process, we aim to exploit this characteristic by inferring underlying impulses.

In this research, we propose a simplified system to understand leptin regulation by exploiting the sparse nature of leptin secretion. If plasma leptin levels are sampled at a 10-minute sampling rate over 24-hours, we obtain a maximum of 40 leptin secretion events out of a total of 1440 secretion events, therefore, the nature being sparse. The sparse system identification of leptin dynamics over time makes it possible to recover the number of leptin pulses. It further allows us to recover information about the pulse amplitudes and intervals of occurrence from collected data. For an extensive understanding of leptin secretion, it is necessary to unveil the pulses originating from the hypothalamus as an outcome to leptin signaling. The pulses resulting in leptin regulation and secretion are the outcome of several contributing factors such as effects of other hormones on leptin regulation, density of leptin receptors, and food intake. Similar to leptin, cortisol secretion is a sparse process. Sparse system identification of cortisol will also lead us to recover the abstraction of secretion events coming from the HPA-axis. It also provides us important insight about the way it is infused by the adrenal glands and cleared by the liver. The proposed architecture has been previously verified for cortisol in healthy subjects and also on patients suffering from chronic fatigue and fibromyalgia syndromes (32–34). Here, we propose to study leptin-cortisol behavior by first observing the underlying pulses and estimating the physiological infusion and clearance rates in the system using two separate regulatory minimal models, one each for cortisol and leptin, and then determining the relationship between them. Similar to the results of previous study by Aschbacher et al. (23), we observe a negative relationship between cortisol and leptin. This relationship is observed based on a negative correlation. To further strengthen this observation, we perform the Granger causality test.

**Figure 1:**
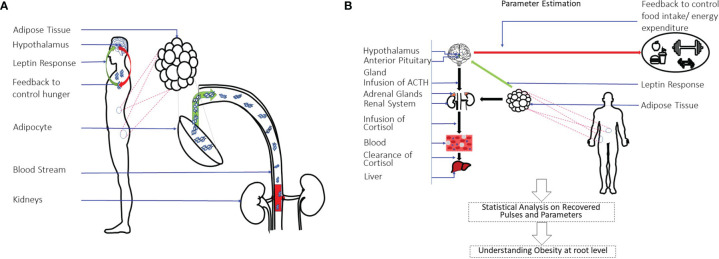
Leptin Regulation Model and an Overview of System-Theoretic Approach. **(A)** Shows the leptin secretion & regulation model. Leptin is the hormone to signal the hypothalamus regarding energy storage and use. It is produced and secreted primarily by adipocytes. The renal system is responsible for clearing it. **(B)** Shows the overall approach used in this study. A state-space model with physiological constraints is designed. A deconvolution approach is then used to estimate the secretion events and the physiological parameters. A statistical analysis is done on the estimations to understand the relationship of leptin-cortisol rhythms and dynamics in detail.

**Figure 1A** shows a pictorial representation of the leptin regulation and secretion model. **Figure 1B** shows a pictorial over all of the approach used in this research. This approach yields three important parameters: the timing and amplitudes of the leptin secretion events, the infusion rate of leptin by the adipose tissue, and the clearance rate by the renal system. We further conduct statistical analysis on the measured and estimated hormone levels. Finally, we propose that this method can be utilized to identify difference between patient and matched control participants for future characterization related to leptin deregulation similar to our previous approach (32).

## 2 Results

We utilize hormone assay from eighteen premenopausal women with mean body mass index (BMI) 33 with range 30–41kgm^-2^; mean age 37.5 with range 22–51 years; at morning (at 9 AM) mean cortisol level 11.02µg/dl and the corresponding standard deviation 3.65 *μg*/*dl*. Th distribution of average leptin level for all subject is 3.3583 ± 1.0633 *μg*/*dl*. The corresponding fat distribution is 39.61 ± 3.08 *kg* and lean body mass distribution is 55.094 ± 6.55 *kg*. Total cholesterol 4.7 ± 0.2 mmol/l (range 3.7–5.8 mmol/l), LDL cholesterol 2.99 ± 1.57 mmol/l (range 2.03– 4.00 mmol/l), and HDL cholesterol 1.54 ± 0.08 mmol/l (range 1.03–2.32 mmol/l). Homeostasis model assessment for insulin resistance (HOMA-IR) was estimated to be 3.25 ± 0.48 with placebo and 2.32 ± 0.19 after treating with Bromocriptine (35). The statistical test between two distribution showed significant difference with *p* = 0.01.

### 2.1 Pulsatile Nature of Leptin

**Figure 2:**
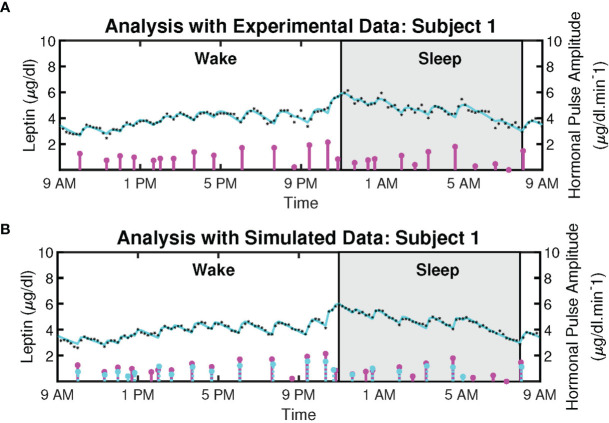
Deconvolved Twenty-Four Hours Leptin Levels in Patient with Obesity. **(A)** Sub-plot shows the measured 24-hour leptin time series (black diamonds), the reconstructed leptin levels (cyan curve), the estimated pulse timings, and amplitudes (magenta vertical lines) for experimental data. **(B)** Sub-plot shows the simulated 24-hour leptin time series (black diamonds), the reconstructed leptin levels (cyan curve), the simulated pulse timings and amplitudes (cyan vertical lines), the recovered pulse timings, and amplitudes (magenta vertical lines) for simulated data.

**Figure 2** shows the example deconvolution results of measured and reconstructed blood leptin levels of women with obesity for both experimental and simulated data for one subject. **Figure 2A** exhibits the estimated amplitudes and timings of hormonal secretory events. It also presents the experimental leptin data and estimates predicted by the model. The amplitude variations of the pulses are due to the circadian rhythm of underlying leptin release, and the variations in the timings are because of the ultradian rhythm (36). The number of recovered secretory events for all subjects were within the physiologically plausible range (i.e., 20-40 events per day). This experimental data includes 18 subjects with obesity. The black diamonds in **Figure 2A** represent the measured leptin levels obtained from blood samples. By performing deconvolution, we obtain the hormonal secretory pulses, which are used to obtain the reconstructed signal. The square of the multiple correlation coefficient (*R* ^2^) is between 0.8327 and 0.98603. *Y*_1_ and *Y*_2_ are the infusion rate of leptin by adipose tissue and the clearance rate of leptin by the renal system, respectively.

**Figure 3:**
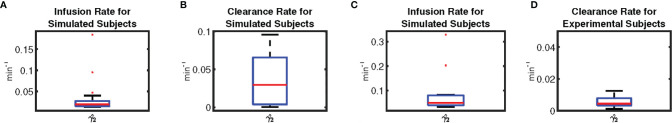
Box-plot of Physiological Parameters of Leptin Levels. Subplots, respectively illustrate the sample distribution of **(A)** the infusion rate for simulated leptin data, **(B)** the clearance rate for simulated leptin data, **(C)** the infusion rate for experimental leptin data, and **(D)** the clearance rate for experimental leptin data, depicting the median (red line), the lower (Q1) to upper (Q3) quartile range (blue rectangle), and 9 to 91 percentile range (black line and black dashed line).

Moreover, we simulated the data to further validate the proposed model. We simulated 18 leptin datasets, each corresponding to an experimental profile. An example of the result is presented in **Figure 2B**. The datasets are simulated from the estimated pulses of the experimental measurements shown in **Figure 2A**. We added Gaussian noise with standard deviation based on interassay co-efficient of variability (σ) provided in (23), defined in the **Supplementary Information**. **Figure 2B** shows an example of the ground truth of the sparse input, the estimated input, and the simulated leptin data. The blue stars show the estimated 24-hour leptin data, the black curve shows the estimated leptin levels, and the vertical blue lines show the amplitudes and timings of the simulated data. The vertical red lines in **Figure 2B** show the amplitudes and timings of the estimated hormone secretory events. **Figure 3** shows the sample distribution for infusion and clearance rates for simulated and experimental leptin levels.

### 2.2 Pulsatile Nature of Cortisol

**Figure 4:**
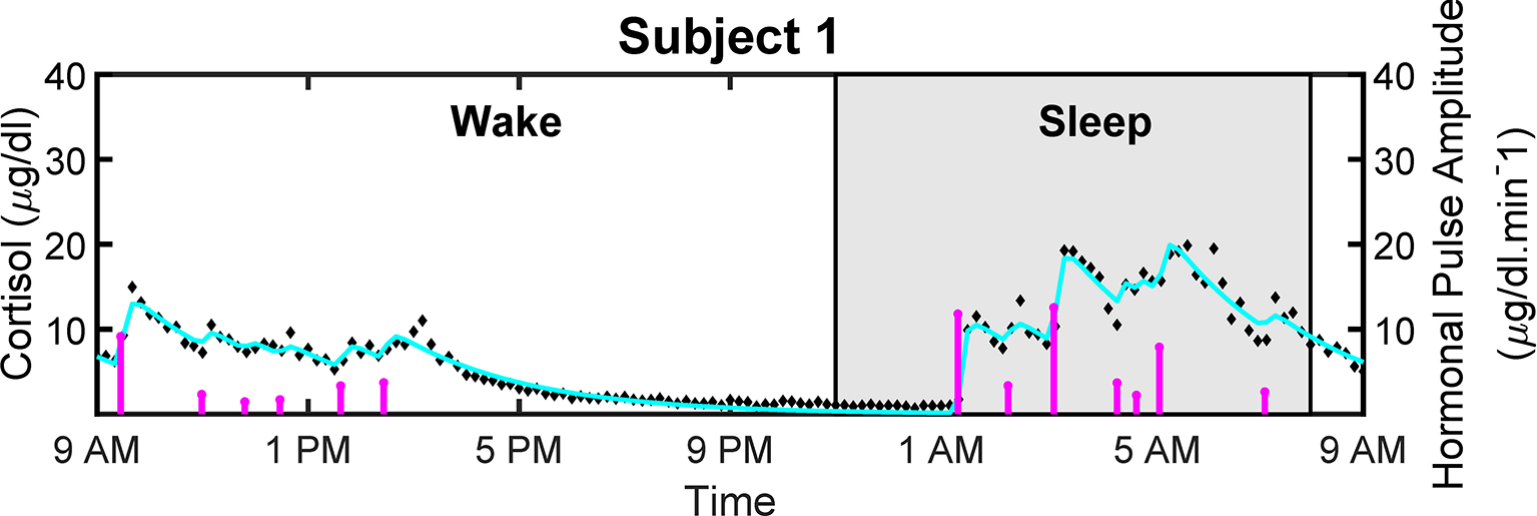
Deconvolved Experimental Twenty-Four Hour Cortisol Levels in Patient with Obesity. Plot shows the measured 24-hour cortisol time series (black diamonds), the reconstructed cortisol levels (cyan curve), the estimated pulse timings and amplitudes (magenta vertical lines).

**Figure 4** shows the measured and reconstructed blood cortisol levels of women with obesity. It shows the measured cortisol levels, reconstructed cortisol levels, and estimated amplitudes and timings of hormonal secretory events. The amplitude and timing variations of the pulses are due to the circadian rhythm and ultradian rhythm of the underlying cortisol release (37). The black diamonds in **Figure 4** represent the measured cortisol levels obtained from blood samples. By performing deconvolution, we obtain the abstraction of hormonal secretory events (blue vertical lines in **Figure 4**), which are used to obtain the reconstructed signal (red curve in **Figure 4**). The square of the multiple correlation coefficient (*R* ^2^) is between 0.87557 and 0.98023. As this approach has been previously examined for cortisol data in different studies, we do not validate it on simulated data (33, 34, 38).

### 2.3 Leptin-Cortisol Antagonism

We seek to test how the leptin hormone samples and cortisol hormone samples are related. In this study, we look at the direct correlation between the sample regardless of other factors. Then, we also perform Granger causality analysis. This way we obtain an overall understanding of the apparent relationship between the leptin and cortisol hormone regardless of the other factors for the current dataset. Consideration of more flexible models that accounts for different factors such as age, gender, BMI, etc., and complexities related to nonlinearity is kept for future work.

#### 2.3.1 Spearman Correlation

We obtain Spearman correlation coefficient to measure the strength of a monotonic association between two variables based on the nonparametric measure of rank correlation. **Table 1** shows the Spearman correlation coefficient between measured leptin levels and measured cortisol levels for 18 subjects. It also shows correlation coefficients for estimated as well as measured leptin and cortisol levels. The outcome for both these cases is very similar and shows that the proposed model retains the properties previously known.

**Table 1:** Comparison of spearman correlation.

Subject	Avg. Cortisol *µg*/*dl*	Avg. Leptin *µg*/*dl*	Coefficient for measured levels	p-value	Coefficient for estimated levels	p-value	BMI	Age
1	7.07	4.19	-0.29	<0.001	-0.28	<0.001	32.7	34
2	7.27	5.19	-0.54	<0.001	-0.57	<0.001	40.5	33
3	6.05	4.72	-0.05	0.5732	-0.06	0.441	30.1	44
4	4.76	4.99	-0.32	<0.001	-0.28	<0.001	38.4	34
5	5.44	2.69	-0.76	<0.001	-0.81	<0.001	33.8	38
6	5.75	2.86	-0.43	<0.001	-0.43	<0.001	30.3	36
7	8.40	2.38	0.28	<0.001	0.27	<0.01	31.0	40
8	7.10	1.47	-0.45	<0.001	-0.43	<0.001	32.2	46
9	7.63	4.02	-0.30	<0.001	-0.26	<0.01	35.1	25
10	7.35	1.94	-0.20	0.0190	-0.22	<0.01	31.8	37
11	5.14	2.95	-0.31	<0.001	-0.30	<0.001	31.1	22
12	5.13	3.83	-0.65	<0.001	-0.69	<0.001	34.3	45
13	4.32	2.96	-0.36	<0.001	-0.37	<0.001	35.3	38
14	5.25	3.97	-0.30	<0.001	-0.33	<0.001	31.4	32
15	6.38	2.20	0.05	0.5888	0.05	0.572	33.3	51
16	4.60	3.83	-0.19	0.0226	-0.20	0.019	31.6	39
17	5.98	2.67	-0.20	0.0136	-0.19	0.022	31.2	43
18	7.07	3.54	-0.16	0.069	-0.15	0.068	32.7	38

Comparing Spearman correlation coefficients between the measured serum leptin and cortisol levels, and the model estimated leptin and cortisol levels.

**Figure 5:**
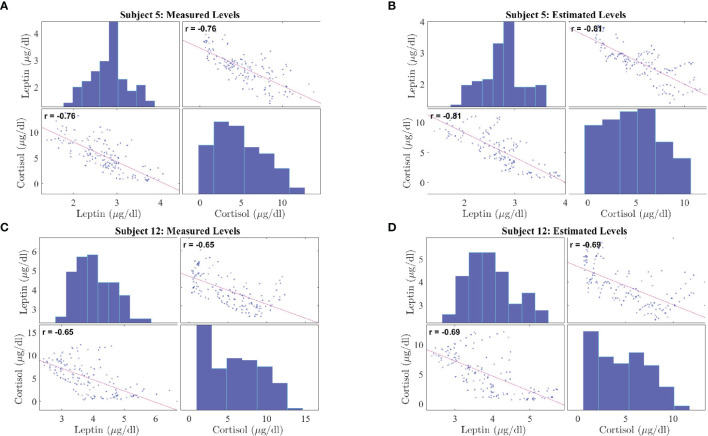
Correlation Plot between Leptin and Cortisol Levels in Patients with Obesity. The correlation plots are as follows: **(A)** measured leptin and cortisol levels for subject 5, **(B)** estimated leptin and cortisol levels for subject 5, **(C)** measured leptin and cortisol levels for subject 12, **(D)** estimated leptin and cortisol levels for subject 5. Each correlation plot incorporates a histogram and a scatter plot: the top left plot shows a histogram depicting leptin distributions and the bottom right plot shows a histogram depicting cortisol distributions, the bottom left plot and top right plot shows the scatter plot depicting correlation between leptin and cortisol.

**Figure 5** shows the correlation plots for leptin and cortisol levels in subjects 5 and 12 who showed the highest negative correlation for both measured and estimated cases. We plot for both the measured and estimated levels to show the similarities. Except for in subjects 3, 15, and 18, we have observed significant correlation (i.e. *p* ≤ 0.05). All other correlation plots are provided in the **Supplementary Information**. This analysis shows, a negative association exists between leptin and cortisol levels across all time points in most, but not all, patients. The negative correlation on the same experimental dataset has been first identified by Aschbacher et al. (23). In this study, we see that the outcome also hold on the reconstructed data from the proposed model and sparse deconvolution.

#### 2.3.2 Granger Causality

**Figure 6:**
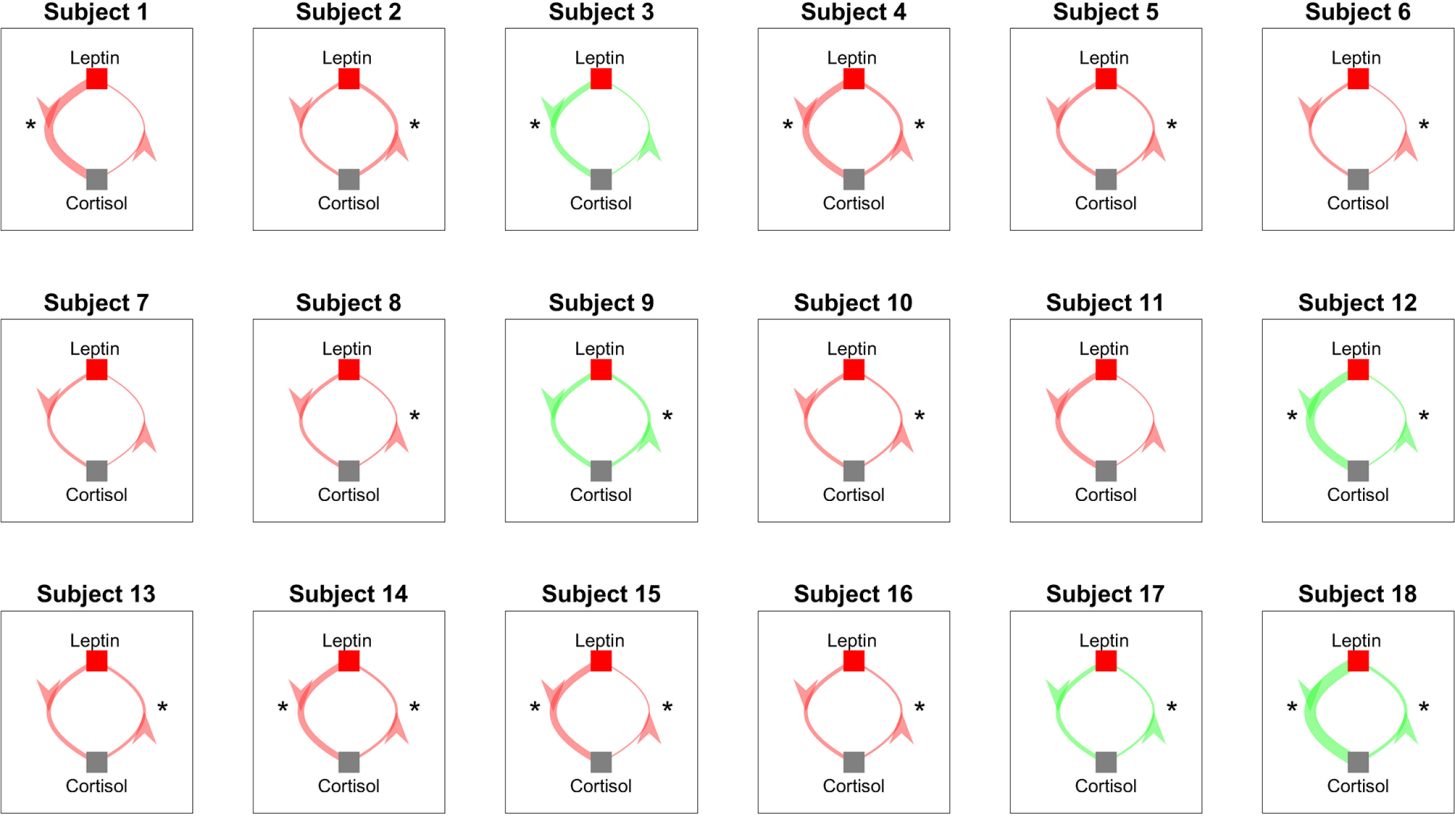
Granger Causality Map Estimated for Leptin and Cortisol for 18 Women with Obesity. Red color shows a negative causal influence and green color shows a positive causal influence, respectively. The direction of each arrow indicates that the variable at the start of the arrow is causing the variable at the end of the arrow. The thickness of the arrows represent the magnitude of the interaction. Starts next to the arrows denote that the corresponding prospective predictions are statistically significant with *p* < 0.05. * denotes statistical significance.

We perform Granger causality analysis to infer the prospective prediction between leptin and cortisol. **Figure 6** shows the results from the Granger causality analysis. Fourteen out of eighteen subjects showed statistically significant causal relationship from cortisol to leptin (i.e., cortisol samples from past can predict the present leptin samples). The corresponding lags are 36.64 ± 21.68 samples. Out of these fourteen subjects, ten showed the negative causal relationship. Seven out of eighteen showed statistically significant causal relationships from leptin to cortisol and four showed statistically significant negative relationships. The corresponding lags are 10.43 ± 17.95 samples. The evidence of possible negative association is in accordance with the previous findings in previous correlation based study by Aschbacher et al. (23).

#### 2.3.3 Secretion Characteristics Based on Between Subject Variability

**Figure 7:**
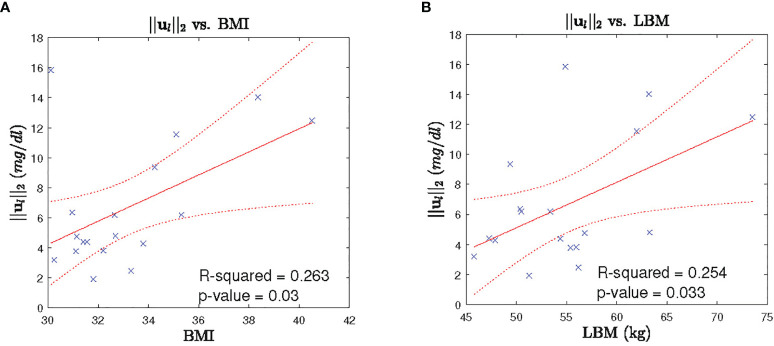
Leptin Secretion Characteristics vs. BMI/LBM. Each panel shows: **(A)** the scatter plot (blue cross) of BMI vs l_2_-norm of leptin secretion u_2_, fitted regression line (red solid line), corresponding 95% confidence interval (dotted red curve), **(B)** the scatter plot (blue cross) of LBM vs l_2_-norm of leptin secretion u_1_, fitted regression line (red solid line), corresponding 95% confidence interval (dotted red curve).

In order to investigate the between subject variability, we perform investigation of the leptin secretion characteristics such as the *l*_2_ -norm of the recovered leptin pulse vector (i.e. || ***u*** *_l_* ||_2_), infusions rates, and clearance rates against the factors such as BMI, lean body mass (LBM), HOMA-IR, and age with regression analysis. Similarly, we investigated the cortisol secretion characteristics such as the *l*_2_-norm of the recovered leptin pulse vector (i.e. || ***u*** *_c_* ||_2_), infusions rates, and clearance rates against the same factors factors. All the investigation were statistically insignificant (*p* > 0) except for the two cases. The regression analysis results between the *l*_2_-norm of leptin secretion (*u*_l_) vs. LBM and the *l*_2_-norm of leptin secretion (*u*_l_) vs. BMI showed statistically significant relationships (*p* < 0.05). **Figure 7** show the scatter plot and the corresponding regression lines.

## 3 Discussion

A complete model for the representation of hormonal variations must include all important intrinsic parameters such as forward and backward linkages between the hypothalamus, adipose tissue, anterior pituitary, adrenal gland, renal system, and liver as well as extrinsic parameters like stress, sleep, light, and food (37). It is a challenge to include all these factors while collecting human data, therefore, we propose this simplified minimal model considering the leptin measurements only. With the proposed minimal system theoratic model we formulate an optimization problem to infer unknown underlying the leptin secretion events as well as the corresponding system parameters. Furthermore, we performed the correlation analysis and the Granger causality analysis to investigate some possible directions to combine both cortisol and leptin in a single model in future. Investigation of secretion dynamics my offer a deeper understanding of the diseased conditions.

Understanding the secretion dynamics of leptin and cortisol in obese subjects and designing a model to understand their irregularities is difficult and challenging because the range of the number of leptin secretion events over a 24-hour period has not been accurately established. Licinio et al. (39) compared the leptin levels of women with obesity against healthy women and found that the concentration of independent pulsatile parameters of leptin, such as pulse duration and frequency, remained consistent between the groups, and that the excessive leptin levels in the persons with obesity were due to only an increased pulse height during secretion. The average number of leptin secretory events was found to be 32.0 ± 1.5 per day (39), within a range of 29 to 39 pulses among the subjects. In another study (40), they reported the average number of leptin pulses to be 30.0 ± 1 with the range of pulses between 21 to 39 over a 24-hour sampling period. These studies give a likely average of around 30 pulses per day of leptin, with a range between 20 to 40 being a possibility. We assumed there to be between 20-40 secretory events during a 24-hour period. Since there is no prior knowledge about the exact range of secretion events in women with obesity, we relax the constraints on the pulse range. We relaxed the upper and lower limits of this problem while taking care of the overfitting problem using Generalized Cross Validation - FOCal Under-determined System Solver (GCV-FOCUSS) to find *λ*. Although the upper limit was set to 50, we obtained less than or equal to 40 pulses for all patients. The proposed approach is applicable for a shorter length of hormone assay than a 24-hour assay, as long as it contains at least one secretion event. Therefore, the samples collected over an hour or more can be utilized to obtain the corresponding secretion event and the system parameters. As it can be seen in **Figure 3**, there is a difference in the distribution of the estimated parameters from the experimental data and the simulated data. This could be an indication of the consideration of a complex model in the optimization problem relative to the number of data-point available. Therefore proposed optimization problem might suffer from overfitting as pointed out in (41). As a future work of this, appropriate probabilistic priors can be applied on the system parameters to prevent such overfitting (42).

Unlike leptin, the mathematical modeling of glucocorticoids such as cortisol is a more widely studied problem. Using the coordinate descent approach, we recover the underlying pulses of cortisol along with the infusion of cortisol into plasma by the adrenal glands and clearance by the liver. After recovering the underlying leptin and cortisol pulses, we check the relation between them. In this research, to find the correlation between cortisol and leptin secretory patterns in women with obesity, we keep the units for both hormones consistent. Both cortisol and leptin measurements were converted to *μg*/*dL*. As compared to cortisol measurements, the leptin measurements have lower levels, making it more challenging to deconvolve. Aschbacher et al. observed a leptin-cortisol antagonism (23). This research further suggests that leptin-cortisol dynamics might provide an indirect or functional biomarker of the neuroendocrine starvation response (23). Based on our statistical analysis, we observe a negative correlation for 14 out of 18 subjects between measured leptin and cortisol levels, as well as a negative correlation between the estimated leptin and cortisol levels. However, as no lag has been considered during the correlation analysis which may explain the inhibition action, the negative correlation in this case may suggest an existence of an underlying factor which is causing both of these hormones to change in opposite direction. Therefore, the proposed simplified minimal model to study cortisol and leptin, and then finding the relationship between them, retains the previous properties. Consequently, exploiting the sparse and pulsatile nature of these hormones provides us with a more coherent insight.

As the distributions of the measured and the estimated samples are not Gaussian, we consider the rank-based Spearman correlation measure. A negative relationship has been observed between leptin and cortisol based on Spearman correlation for most of the subjects. The negative correlation between the reconstructed leptin and cortisol data also demonstrate that the proposed model and the corresponding sparse deconvolution also preserves the negative association. To further verify the interdependence of leptin and cortisol dynamics, we investigate the causal relationship between them. We perform the Granger causality analysis on leptin and cortisol to obtain the directional relationship. We observe a negative relationship, i.e., leptin-cortisol antagonism in 14 out of 18 subjects. leptin inhibits the HPA axis, which, *via* neuroendocrine hormones like ACTH, results in a reduction in cortisol (43, 44). Cortisol increases or stimulates production of leptin in adipose tissue (44). It is possible that this relationship may help explain behavioral contributors to obesity such as “stress-eating.” In the remaining 4 subjects, we do not observe an antagonism. It is possible that there exists a potential leptin resistance (45–48) in these subjects. Leptin resistance is a condition in which, even though the body has enough high leptin levels, receptors are desensitized leading to a “low signal.” Consequently, the brain may respond as if it were starving leading to a decrease in postprandial satiety. It is also possible that, due to a faulty leptin signaling, the brain is unable to realize the leptin levels in the body and produce high leptin levels during high cortisol levels. On the other hand, persistent higher concentration of leptin level plasma can also lead to leptin resistance (45, 47). Izquierdo et al. (47), also stated that obese people have high leptin levels and that the treatment of leptin resistance is still a great challenge in the treatment of obesity. Therefore, negative causation further provides a strong reason to study the negative relationship between leptin-cortisol. Furthermore, for some participants, the negative relationship is statistically not significant, while for other participants the relationship is positive. The result shows an indication of individual differences in leptin-cortisol regulation. Moreover, there are studies that did not find any relationship between cortisol and leptin in different groups such as children and adolescents investigating the relation in a single morning measurement of serum cortisol (49). Therefore, it would be very interesting to study these individual and group differences which will be crucial to develop more personalized obesity treatment programs. In other words, if there are differences in the hormone regulation dynamics in presence of obesity, these differences should be studied more thoroughly to before any intervention. This study has limitations in addition to observed variability including lack of measurement of insulin and ACTH, no control group without obesity, no data on the participants’ eating behaviors/physical activity/metabolism, use of Granger causality test, etc. All these factors should be also considered for understanding before considering any treatment program. Personalize treatment programs can be based on many parameters and preferences from patients, which can include possible existence of leptin resistance. That way personalized programs might benefit from taking the leptin cortisol dynamics and possible leptin resistance into account. Although the results obtained from Granger causality test provide some lights towards possible explanation, a conclusive understanding is still elusive which require further future investigation. The challenges includes limited number of subjects and observed between-subject variability. Further investigation is required with consideration of more related factors with larger population.

The leptin regulation model used in this research may be further be useful to understand the regulation dynamics in different symptoms such as leptin congenital deficiency, Alzheimer’s disease, and metabolic syndrome (50, 51). The advantage of using a model based on human physiology is that it can be used to further isolate the organs or tissues responsible for causing a particular deficiency. Determining a system model for the relationship between leptin and cortisol concentrations provides us with a more comprehensive understanding of the biological system’s behavior. This model can provide insight into the effects of the relationship of hormones on weight gain. There exist several bioscience and biosystems technologies used presently to simplify laboratory procedures and ease human life (52, 53). Similar to those available to obtain insulin levels in the human body (54), there will be bioscience technologies that would be able to obtain leptin levels in the human body in the near future.

Similar to leptin and cortisol, skin conductance responses are sparse. Wickramasuriya et al. presents a method to relate internal arousal state to the changes in skin conductance responses (55). Wickramasuriya et al. employ a deconvolution algorithm along with the Bayesian estimation to estimate the hidden arousal state. Azgomi et al. use control technique methods to close the loop and regulate the estimated arousal state within a desired range (56). Furthermore, Wickramasuriya et al. utilize the cortisol observations to track energy regulation in Cushing’s patients (57). Azgomi et al. employ the model presented in (57) to design a control system for closing the loop and regulating energy state in patients with hypercortisolism (58). Similarly, by deconvolving leptin levels and obtaining the hormonal pulses, one may develop a framework to obtain hunger states and regulate it. In another study, researchers in (57) have revealed the fact that cortisol secretion in healthy humans has a circadian nature (57). They derive multi-day data and observe differences between healthy humans and patients. A similar study can be done for leptin since leptin secretion is also influenced by the circadian rhythm. Similarly, another possible outcome of this study is to understand the leptin regulation behavior in women with obesity against their matched healthy subjects. Pednekar et al. (32) uses deconvolution to obtain the underlying hormonal pulses from cortisol data for fibromyalgia, chronic fatigue syndrome, and their matched healthy subjects. The healthy subjects are matched to the patients based on several factors such as age, sex, and weight. The comparison between the parameters and pulses obtained from the patients against their matched healthy subjects showed significant differences in clearance rates and hormonal pulses. A similar study can be done in the future, using leptin data in women with obesity. As another future direction, the model could be further extended to include ACTH and study the differences in hormonal secretory events in women with obesity with respect to healthy subjects (38). These hormonal secretory events are an abstraction of their pulsatile nature and provide a good estimation of the pulses originating in the CNS. A thorough investigation of these events could further help us to understand the role of the CNS as well as kidneys and liver in causing obesity.

In this study, we have proposed a state-space model for describing sparse leptin hormone dynamics. Furthermore, we have proposed a coordinate descent approach for the sparse deconvolution of the sampled leptin data to obtain the pulsatile secretion events. The deconvolution framework also provides estimates of the state-space model parameters. The successful modeling of leptin is the first step to a more comprehensive understanding of dynamics and its interaction with cortisol. Our analysis with Spearman correlation and Granger causality has provided some evidence of negative relation between leptin and cortisol. However, some variability has also been observed which prevents us from concluding a concrete explanation. A more thorough investigation is required as the study has limitation such as lack of including insulin and ACTH measurement, no control group without obesity, no data on the participants’ eating behaviors/physical activity/metabolism, use of Granger causality test that only consider two factors, a small number of participants, low number of samples, etc.

## 4 Methods

### 4.1 Experimental Dataset

In this research, we utilize blood sample data collected from eighteen premenopausal women with obesity for our analysis of leptin and cortisol pulse recovery (59). The participants have given informed consent for participation in the study. They collected data for 18 premenopausal women with obesity over a 24-hour period with 10-minute sampling frequency. All participants were required to have regular menstrual cycles. All studies were done in the early follicular phase of the menstrual cycle. The subjects were within the age group of 22-51 and with BMI between 30-41 kg/m^2^. Subjects were admitted to the research center at 7:00 after an overnight fast. After the subjects rested for 45 min, indirect calorimetry was performed using a ventilated hood for 30 min. Thereafter, a cannula for blood sampling was inserted into an antecubital vein, which was attached to a three-way stopcock and kept patent by a continuous 0.9% NaCl and heparin (1 U/ml) infusion (500 ml/24 h). Each subject followed a consistent schedule with breakfast, lunch, and dinner provided, and had an 8.5 hour sleeping period between 23:00 and 7:30. The blood samples were collected without waking the subjects during this period. Mean fasting glucose concentration was 5.0 ± 0.1 mmol/l (range 4.2– 6.3 mmol/l), insulin 15.3 ± 1.7 mU/l (range 7–28 mU/l), glycosylated hemoglobin (HbA1C) 4.7 ± 0.1% (range 3.9 –5.3%). The above study was approved by the Medical Ethics Committee of Leiden University (23). Subjects were required to be free of chronic disease, and exclusion criteria were fixed, such as shift work, depression, alcohol abuse, and oral contraceptives. The blood samples were assayed every 10 minutes for cortisol and every 20 minutes for leptin and intermediate points were interpolated. Plasma leptin concentrations were estimated by radioimmunoassay with a detection limit of 0.5 *ng*/*liter*, and interassay coefficient of variation of 3.6% to 6.8%. Plasma cortisol was measured with a radioimmunoassay with a detection limit is 25 *nmol* ^–1^ and the intra-assay coefficient of variation ranges between 2% and 4%. A detailed description of the experiment is provided in (23, 59). In this research, we analyze the leptin and cortisol abstraction pulses for 18 subjects obtained from this dataset.

### 4.2 Leptin Model Formulation

The state-space model introduced in this research considers the first-order differential system of equations for leptin synthesis in the adipose tissue and clearance by the renal system. In a 24-hour period, the blood samples were collected at a 10-minute interval, i.e., 144samples in a day. However, leptin was assayed every 20 min and concentrations was obtained for every 10 min by interpolation. If we assume that leptin secretion can happen in every minutes then there are 1440 possible events for 24-h duration. In a discrete space of 1440 events, we observe between 20 to 40 pulses making it sparse in nature. In this model, we intend to utilize the sparse nature of the hormonal secretory events along with other physiological constraints in a state-space model to estimate the amplitude and frequency of hormonal secretory events. The rate of change of leptin concentration in the adipose tissue is equal to the difference between leptin synthesis rate and the leptin infusion rate from adipose tissue into the blood. Similarly, the rate of change of leptin concentration in the serum is equal to the difference between the leptin infusion rate from adipose tissue into the blood and the leptin clearance rate by the renal system (60). The leptin secretion dynamics are represented as follows:


(1) $$\frac{dx_{1}(t)}{dt}=-\gamma_{1}x_{1}(t)+u_{l}(t)\text{ (Adipose Tissue)}$$

(2) $$\frac{dx_{2}(t)}{dt}=+\gamma_{1}x_{1}(t)-\gamma_{2}x_{2}(t)\text{ (Plasma)}$$

where *x*_1_(*t*) and *x*_2_(*t*) represent the effective leptin concentrations inside adipose tissue and leptin concentration in the blood serum, respectively. The model parameters *Y*_1_ and *Y*_2_ represent the infusion rate of leptin by the adipose tissue and the clearance rate of leptin by the kidneys, respectively. Input *u_l_* (*t*) represents the abstraction of effective pulses mainly produced by the adipose tissue. *u_l_* (*t*) can be modeled as a summation of delta functions with $u_{l}(t)=\Sigma_{i=1}^{N}\;q_{i}\;\delta (t-\tau_{i})$ where *q_i_* is the magnitude of the pulse initiated at time *τ_i_*. If no pulse occurs at *τ_i_*, *q_i_* will equal zero. The pulses are assumed to occur at integer minutes, i.e., in a 24-hour period, there are 1440 distinct locations (*N* = 1440). The blood samples are collected every 10 minutes for *M* samples (*M* = 144). Blood samples were collected beginning at $y_{l_{o}}$ and then assayed for leptin. Let $y_{t_{10}},y_{t_{20}},\cdots ,y_{t_{10M}},t_{k}:k=10,20,\ldots ,10M$ . Furthermore, $y_{l_{o}}=y_{t_{10}}$ .


(3) $$y_{l_{t_{k}}}=x_{2}(t_{k})+v_{t_{k}}$$

where $y_{t_{k}}$ and $v_{t_{k}}$ represent the observed leptin level and hormone measurement error at a time *t_k_*, respectively. We model $v_{t_{k}}$ random variables as Gaussian random variable. Then for the system output, we assume that the observed output value at time *t_k_* will be equal to the previous value, y_0_, multiplied by a decay term and added to a secretion value and the error term. We can represent the solution for every time point *t_k_* with the following equation:


(4) $$y_{l_{t_{k}}}={\text{a}}_{t_{k}}y_{l_{0}}+b_{t_{k}}{\text{u}}_{l}+v_{t_{k}}$$

where ${\text{b}}_{{\text{t}}_{\text{k}}}={\left[\frac{\gamma_{1}}{\gamma_{1}+\gamma_{2}}(e^{-\gamma_{2}k}-e^{-\gamma_{1}k})\frac{\gamma_{1}}{\gamma_{1}-\gamma_{2}}(e^{-\gamma_{2}(k-1)}-e^{-\gamma_{1}(k-1)})\;\cdots \;\frac{\gamma_{1}}{\gamma_{1}-\gamma_{2}}(e^{-\gamma_{2}}-e^{-\gamma_{1}})\;\underset{N-k}{\underset{\backslash underbrace\backslash }{\begin{array}{ccc} 0 & \cdots & 0 \end{array}}}\right]}^{'}$ and $a_{t_{k}}=e^{-\gamma_{2}k}$ . ***u*** *_l_* represents the input over the entire 24-hour sampling period, with values *q_i_* over *i* = 1,…,1440. $v_{t_{k}}$ stands for the vector representation of the random variable $v_{t_{k}}$ . We then solve for ${\text{a}}_{t_{k}}$ and $b_{t_{k}}$ using a forced solution approach, multiplying each side of the equation by *e* ^–^ *^at^* and using mathematical methods to obtain the solutions.

From these matrices, we can form a combined representation for the system at any time, using


$${\text{y}}_{l}=[y_{t_{10}}\;y_{t_{20}}\;\cdots y_{t_{10M}}]\prime ,\;\gamma =[\gamma_{1}\;\gamma_{2}]\prime ,\;A_{\gamma }=[a_{t_{10}}\;a_{t_{20}}\;\cdots \;a_{t_{10M}}]\prime ,\;B_{\gamma }=[b_{t_{10}}\;b_{t_{20}}\;\cdots \;b_{t_{10M}}]\prime ,{\text{u}}_{l}=[q_{1}\;q_{2}\;\cdots q_{N}]\prime ,\;\text{and}\;\text{v}=[v_{t_{10}}\;v_{t_{20}}\;\cdots \;v_{t_{10M}}]\prime$$

$$A_{\gamma }=[a_{10}\;a_{20}\ldots a_{10n}]\prime ,\beta_{\gamma }=[b_{10}\;b_{20}\ldots b_{10n}]\prime ,$$

$$\gamma =[\gamma_{1}\;\gamma_{2}]\prime$$

We then represent the system output as:


$${\text{y}}_{l}={\text{A}}_{\gamma }y_{l_{0}}+B_{\gamma }{\text{u}}_{l}+{\text{v}}_{l}$$

Where output vector *y_l_* is dependent on the initial signal value, the input pulses, and the error term. The values of matrices *A*_γ_ and *B*_γ_ are dependent on the values of *y* for the given subject.

### 4.3 Cortisol Model Formulation

In this research, we use the cortisol secretion model provided by Faghih et al. (33). It exploits the sparse nature of hormonal secretory events and other physiological constraints to estimate the amplitude and timings of the secretory events using a state-space model. The rate at which cortisol concentration changes in the adrenal glands is equal to the difference between the rate at which it is infused in the blood and the rate at which it is secreted. The rate of change of serum cortisol concentration is equal to the difference between the rate at which it is infused by the adrenal gland into the blood and the rate at which it is cleared by the liver from the blood. The cortisol secretion dynamics are represented as follows:


(5) $$\frac{dx_{3}(t)}{dt}=-\psi_{1}x_{3}(t)+u_{c}(t)\;(\text{Adrenal Glands})$$

(6) $$\frac{dx_{4}(t)}{dt}=\psi_{1}x_{3}(t)-\psi_{2}x_{4}(t)\;(Serum)$$

where *x*_3_(*t*) and *x*_4_(*t*) represent the cortisol concentrations in the adrenal glands and the blood serum, respectively. The physiological model parameters *ψ*_1_ and *ψ*_2_ represent the infusion rate of cortisol by the adrenal glands and the cortisol clearance rate by the liver respectively. Input *u_c_* (*t*) represent the abstraction of pulses coming from the hypothalamus responsible for the production of cortisol. *u_c_* (*t*) can be modeled as a summation of delta functions with $u_{c}(t)=\Sigma_{i=1}^{N}\;p_{i}\;\delta (t-\tau_{i})$ . *p_i_* is the magnitude of the pulse initiated at time *τ_i_*. If no pulse occurs at *τ_i_*, *q_i_* will equal zero. The pulses are assumed to occur at integer minutes, i.e. in a 24-hour period, there are 1440 distinct locations (*N* = 1440). The blood samples are collected every 10 minutes, for *M* samples (M = 144). As explained in Section 4.2 the measurement output can be represented as:


$$y_{c_{t_{k}}}=x_{4}(t_{k})+\omega_{t_{k}}$$

where $y_{t_{k}}$ and $v_{t_{k}}$ are the observed cortisol concentration and the measurement error, respectively. The initial concentration of cortisol in adrenal glands and serum is assumed to be zero and $y_{c_{o}}$ . The system is further expressed as:


$$y_{c}=A_{\psi }y_{c_{0}}+B_{\psi }u_{c}+\omega_{c}$$

where


$$\begin{array}{l} y_{c}=[y_{t_{10}}\;y_{t_{20}}\;\cdots \;y_{t_{10M}}]\prime ,\cdot \psi =[\psi_{1}\;\psi_{2}]',\cdot A_{\psi }=[a_{t_{10}}\;a_{t_{20}}\;\cdots \;a_{t_{10M}}]',\cdot B_{\psi }=[b_{t_{10}}\;b_{t_{20}}\;\cdots \;b_{t_{10M}}]',\;u_{c}=[q_{1}\;q_{2}\cdots q_{N}]',\omega_{c}=[w_{t10}\;w_{t20}\cdots b_{t_{10M}}]',\\ a_{t_{i}}=e^{e-\psi_{2}i}\;b_{t_{i}}=[\frac{\psi_{1}}{\psi_{1}-\psi_{2}}(e^{-\psi_{2}i}-e^{-\psi_{1}i})\;\frac{\psi_{1}}{\psi_{1}-\psi_{2}}(e^{-\psi_{2}i(i-1)}-e^{-\psi_{1}i(i-1)})\;\cdots \;\frac{\psi_{1}}{\psi_{1}-\psi_{2}}(e^{-\psi_{2}}-e^{-\psi_{1}})\;\underset{N-i}{\underset{\backslash underbrace\backslash }{0\;\cdots \;0}}]'. \end{array}$$

#### 4.3.1 Granger Causality Analysis

We utilize Granger Causality to determine the prospective prediction between leptin and cortisol. Let $y_{c_{t_{k}}}$ and $y_{l_{t_{k}}}$ are two stationary processes sampled every 10 minutes. A simple causal model for the interaction between leptin and cortisol is given as follows (61):


(7) $$y_{l_{t_{k}}}=\sum_{j=1}^{m_{l_{2}}}\alpha_{l,j}y_{l_{t_{k^{-10j}}}}+\sum_{j=1}^{m_{l_{2}}}\beta_{l,j}y_{c_{t_{k^{-10j}}}}+\in_{l_{t}}$$

(8) $$y_{c_{t_{k}}}=\sum_{j=1}^{m_{c_{1}}}\alpha_{c,j}y_{c_{t_{k^{-10j}}}}+\sum_{j=1}^{m_{c_{2}}}\beta_{c,j}y_{l_{t_{k^{-10j}}}}+\in_{c_{t}}$$

According to the definition of the causality, $y_{c_{t_{k}}}$ is causing $y_{l_{t_{k}}}$ provided some *β_l,j_* is not zero and $y_{l_{t_{k}}}$ is causing $y_{c_{t_{k}}}$ if some *β_c,j_* is not zero. If both *β_c,j_* and *β_l,j_* are not zero, there is exist a feedback relationship between $y_{c_{t_{k}}}$ and $y_{l_{t_{k}}}$ . Here, *ha_j,j_* and *α_c,j_* represents the coefficients related to the self interaction. $\in_{l_{t}}$ and $\in_{c_{t}}$ represent the model errors. We model them as i.i.d Gaussian random variables.

We use the Bayesian information criteria (BIC) to find $m_{l_{1}}$ and $m_{c_{1}}$ considering all *β_l,j_* = *β_c,j_* = 0. In contrast, we find the $m_{l_{2}}$ and $m_{c_{2}}$ using BIC considering $m_{l_{1}}$ and $m_{c_{1}}$ numbers of nonzero *α_l,j_* and *α_c,j_* in (7) and (8), respectively. We first fit the models in (7) and (8) to the data with *β_l,j_* = *β_c,j_* = 0. We obtain the likelihoods as $L_{l}(y_{l_{t_{k}}})$ and $L_{c}(y_{c_{t_{k}}})$ . Then, we assume *β_l,j_*, *β_c,j_* ≠ 0 and fit the model to the data. We obtain the corresponding likelihood $L_{l}(y_{l_{t_{k}}}|y_{c_{t_{k}}})$ and $IL_{c}(y_{c_{t_{k}}}|y_{l_{t_{k}}})$ . Therefore, the Granger causality depicts how cortisol is causing leptin,


$$LLR_{l,c}=\text{sign}\left(\sum_{j=1}^{m_{l_{2}}}\beta_{l,j}\right)\times \left(-\log\frac{L_{l}(y_{l_{t_{k}}})}{L_{l}(y_{l_{t_{k}}}|y_{c_{t_{k}}})}\right)$$

and how leptin is causing cortisol,


$$LLR_{c,l}=\text{sign}\left(\sum_{j=1}^{m_{c_{2}}}\beta_{c,j}\right)\times \left(-\log\frac{L_{l}(y_{l_{t_{k}}})}{L_{l}(y_{l_{t_{k}}}|y_{c_{t_{k}}})}\right)$$

where negative sign denotes inhibitory, positive sign denotes excitatory, and zero means no prospective prediction. The magnitude gives the relative strength of the prospective prediction. Furthermore, we calculate the F-statistics for testing whether the prospective prediction is statistically significant or not (61).

## Data Availability Statement

The original contributions presented in the study are included in the article/**Supplementary Material**. Further inquiries can be directed to the corresponding author.

## Ethics Statement

The studies involving human participants were reviewed and approved by Medical Ethics Committee of Leiden University. The patients/participants provided their written informed consent to participate in this study.

## Author Contributions

RF conceived and designed the study. MA and DP performed the research, analyzed data and wrote the manuscript. RF and MA developed the algorithms and analysis tools. All authors revised the manuscript. All authors contributed to the article and approved the submitted version.

## Funding

This work was supported in part by NSF grant 1942585 - CAREER: MINDWATCH: Multimodal Intelligent Noninvasive brain state Decoder for Wearable AdapTive Closed-loop arcHitectures and 1755780 - CRII: CPS: Wearable-Machine Interface Architectures. RF as the senior author. NYU faculty start-up funds covered the article processing charges.

## Conflict of Interest

The authors declare that the research was conducted in the absence of any commercial or financial relationships that could be construed as a potential conflict of interest.

## Publisher’s Note

All claims expressed in this article are solely those of the authors and do not necessarily represent those of their affiliated organizations, or those of the publisher, the editors and the reviewers. Any product that may be evaluated in this article, or claim that may be made by its manufacturer, is not guaranteed or endorsed by the publisher.
