# Corrigendum: Sparse system identification of leptin dynamics in women with obesity

**DOI:** 10.3389/fendo.2022.1078146

**Published:** 2022-12-14

**Authors:** Md. Rafiul Amin, Divesh Deepak Pednekar, Hamid Fekri Azgomi, Herman van Wietmarschen, Kirstin Aschbacher, Rose T. Faghih

**Affiliations:** ^1^ Department of Electrical and Computer Engineering, University of Houston, Houston, TX, United States; ^2^ Department of Nutrition and Health, Louis Bolk Instituut, Bunnik, Netherlands; ^3^ Department of Psychiatry, Weill Institute for Neurosciences, University of California, San Francisco, San Francisco, CA, United States

**Keywords:** obesity, leptin, cortisol, state-space, sparse recovery, deconvolution, correlation, grange-causality

In the published article, there were two errors. The errors are in two of the equations,

A correction has been made to Methods, Leptin Model Formulation, Paragraph 1, Equation (2). This sentence (equation) previously stated:


(2) $$\frac{dx_{2}(t)}{dt}=-\gamma_{1}x_{1}(t)+\gamma_{2}x_{2}(t)\;(plasma)$$

The corrected sentence (equation) appears below:


(2) $$\frac{dx_{2}(t)}{dt}=+\gamma_{1}x_{1}(t)-\gamma_{2}x_{2}(t)\;(plasma)$$

A correction has been made to Methods, Cortisol Model Formulation, Paragraph 1, Equation (6). This sentence (equation) previously stated:


(6) $$\frac{dx_{4}(t)}{dt}=\psi_{1}x_{3}(t)+\psi_{2}x_{4}(t)\;(Serum)$$

The corrected sentence (equation) appears below:


(6) $$\frac{dx_{4}(t)}{dt}=\psi_{1}x_{3}(t)-\psi_{2}x_{4}(t)\;(Serum)$$

The original article has been updated.

