# Supplementary material for: Sparse System Identification of Leptin Dynamics in Women With Obesity
Source: Front Endocrinol (Lausanne). 2022 Apr 5;13:769951. doi: 10.3389/fendo.2022.769951 (PMC9037068; doi:10.3389/fendo.2022.769951)
Supplement: Supplementary file 1 [file DataSheet_1.pdf]

1 Supplementary Information: Results

1.1 Pulsatile Nature of Leptin:

Figure 1-a shows the measured and reconstructed blood leptin levels of women with obesity for experimental data. It exhibits the estimated amplitudes and timings of hormonal secretory events, experimental leptin data, and estimates predicted by the model. The amplitude variations of the pulses, that are due to the circadian rhythm of underlying leptin release and the variations in the timings, are because of the ultradian rhythm[1]. The number of recovered secretory events for all the subjects were within the physiologically plausible range. This experimental data includes 18 women with obesity. The black diamonds in Figure 1-a represent the measured leptin level obtained from blood samples. By applying the deconvolution algorithm, we obtain the hormonal secretory pulses, which are used to find the reconstructed signal. The square of the multiple correlation coefficient (R^2) is between 0.8327 and 0.98603. γ_1 and γ_2 are the infusion rate of leptin by adipose tissue and the clearance rate of leptin by renal system in experimental subjects, respectively. $\hat{\gamma}_1$ and $\hat{\gamma}_2$ are infusion rate and clearance rate of leptin in simulated subjects, respectively. The number of recovered pulses for the experimental data is between the pulse range 20-40.

In this research, we simulated data to further validate the proposed model. We simulated 18 leptin datasets, each corresponds to an experimental dataset. The datasets are simulated from the estimated pulses of the experimental dataset shown in Figure 1-b. We added Gaussian noise with standard deviation based on interassay co-efficient of variability (σ) provided in[2]. Figure 1-b shows the ground truth of the sparse input, the estimated input, and the simulated leptin data. The blue stars show the estimated 24-hour leptin data, the black curve shows the estimated leptin levels, the vertical blue lines show the amplitudes and timings of the simulated data, and the vertical red lines show the amplitudes and timings of estimated hormone secretory events. Table 1 shows the estimated number of pulses, physiological model parameters, R^2, the percentage error in estimating the parameters, and the standard deviation of Gaussian noise added for simulation of the datasets.

1.2 Pulsatile Nature of Cortisol:

Figure 2 shows the measured and reconstructed blood cortisol levels of women with obesity. It shows the measured cortisol levels, reconstructed cortisol levels, estimated amplitudes, and timings of hormonal secretory events for the subjects. The amplitude and timing variations of the pulses are due to the circadian rhythm and ultradian rhythm of underlying cortisol release[3]. The black diamonds in Figure 2 represent the measured cortisol level obtained from blood samples. By applying the deconvolution algorithm, we obtain the abstraction of hormonal secretory events (blue vertical lines in Figure 2), which are used to derive the reconstructed signal (red curve in Figure 2). The square of the multiple correlation coefficient (R^2) is between 0.87557 and 0.98023. Since this approach has been previously used for cortisol in different studies and already been validated before, we do not further validate it on simulated data[4–6].

Table 1. The Estimated Model Parameters and the Squares of the Multiple Correlation Coefficients (R^2) for the Simulated Leptin Time Series.

Subject	Infusion Rate ($\hat{\gamma}_1$)	Clearance Rate ($\hat{\gamma}_2$)	Number of Pulses (N)	R^2	Interassay CV (σ)	$\frac{(\gamma_1-\hat{\gamma}_1)}{\gamma_1}(\%)$	$\frac{(\gamma_2-\hat{\gamma}_2)}{\gamma_2}(\%)$
1	0.1841	0.0032	32	0.9968	0.2158	10.55	14.38
2	0.0952	0.0655	30	0.9983	0.2467	11.61	7.02
3	0.0402	0.0484	32	0.9952	0.1825	26.81	15.92
4	0.0145	0.0104	23	0.9974	0.2994	1.40	1.40
5	0.0146	0.0400	21	0.9906	0.1396	8.96	8.96
6	0.0135	0.0201	22	0.9905	0.1162	6.30	6.30
7	0.0192	0.0734	26	0.9744	0.1186	8.13	6.08
8	0.0274	0.0038	27	0.9560	0.0801	11.38	2.13
9	0.0260	0.0770	35	0.9986	0.2227	12.75	15.12
10	0.0472	0.0023	29	0.9744	0.0927	7.99	14.13
11	0.0193	0.0631	28	0.9903	0.1514	8.29	6.85
12	0.0205	0.000411	28	0.9980	0.2212	18.00	24.2
13	0.0189	0.0389	26	0.9958	0.1276	10.84	9.09
14	0.0129	0.0143	21	0.9938	0.2215	10.26	10.26
15	0.0142	0.000240	28	0.9557	0.1149	5.33	5.33
16	0.0208	0.0105	26	0.9955	0.2338	6.67	0.00
17	0.0153	0.0864	28	0.9847	0.1460	3.37	18.06
18	0.0180	0.0955	24	0.9944	0.1578	9.76	1.29

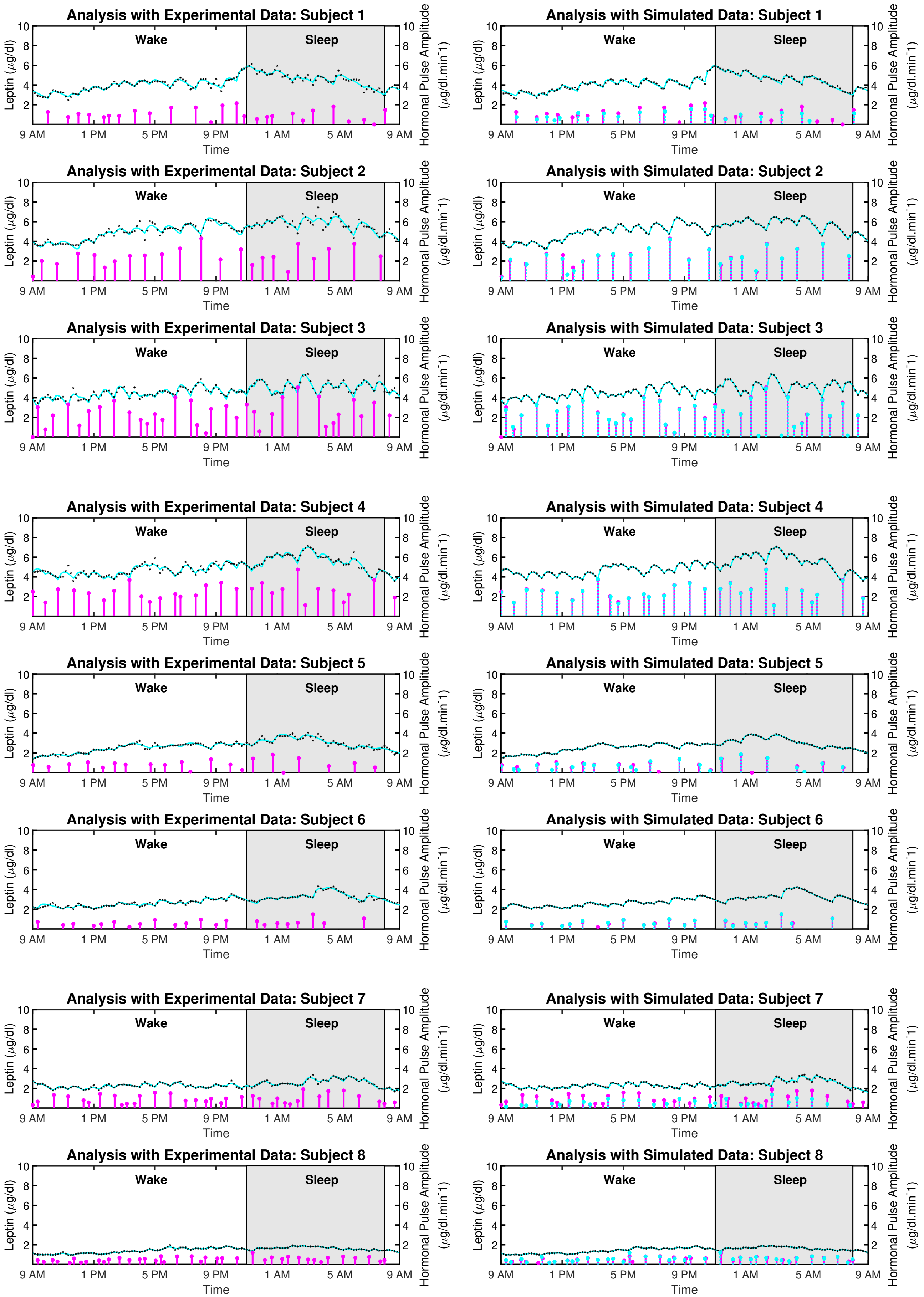

Analysis with Experimental Data: Subject 1
Analysis with Simulated Data: Subject 1
Analysis with Experimental Data: Subject 2
Analysis with Simulated Data: Subject 2
Analysis with Experimental Data: Subject 3
Analysis with Simulated Data: Subject 3
Analysis with Experimental Data: Subject 4
Analysis with Simulated Data: Subject 4
Analysis with Experimental Data: Subject 5
Analysis with Simulated Data: Subject 5
Analysis with Experimental Data: Subject 6
Analysis with Simulated Data: Subject 6
Analysis with Experimental Data: Subject 7
Analysis with Simulated Data: Subject 7
Analysis with Experimental Data: Subject 8
Analysis with Simulated Data: Subject 8
Wake
Sleep
Leptin (μg/dl)
Hormonal Pulse Amplitude (μg/dl.min^-1)
Time
9 AM
1 PM
5 PM
9 PM
1 AM
5 AM
9 AM

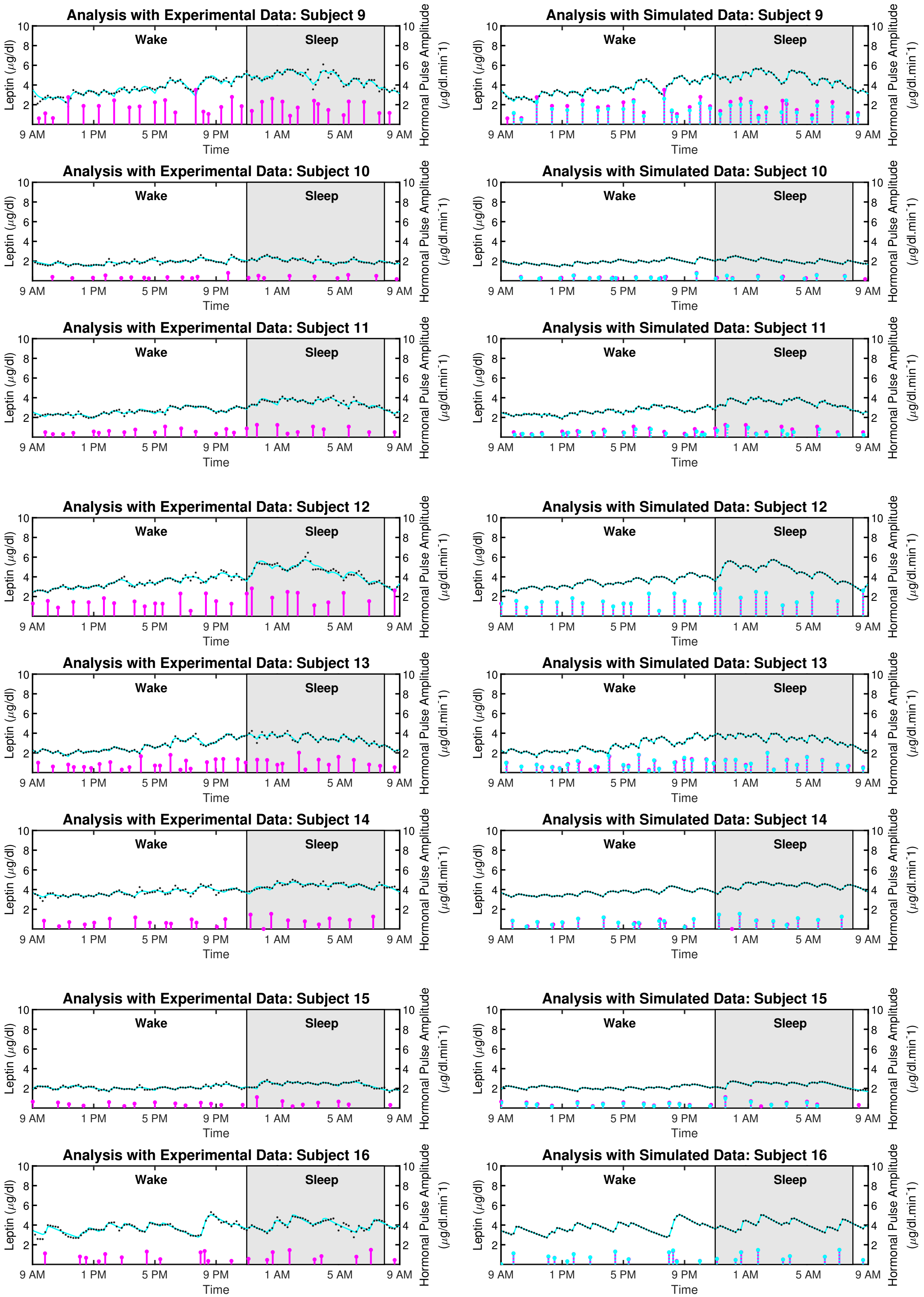
Analysis with Experimental Data: Subject 9
Analysis with Simulated Data: Subject 9
Analysis with Experimental Data: Subject 10
Analysis with Simulated Data: Subject 10
Analysis with Experimental Data: Subject 11
Analysis with Simulated Data: Subject 11
Analysis with Experimental Data: Subject 12
Analysis with Simulated Data: Subject 12
Analysis with Experimental Data: Subject 13
Analysis with Simulated Data: Subject 13
Analysis with Experimental Data: Subject 14
Analysis with Simulated Data: Subject 14
Analysis with Experimental Data: Subject 15
Analysis with Simulated Data: Subject 15
Analysis with Experimental Data: Subject 16
Analysis with Simulated Data: Subject 16
Wake
Sleep
Leptin (μg/dl)
Hormonal Pulse Amplitude (μg/dl.min^-1)
Time
9 AM
1 PM
5 PM
9 PM
1 AM
5 AM
9 AM
2
4
6
8
10

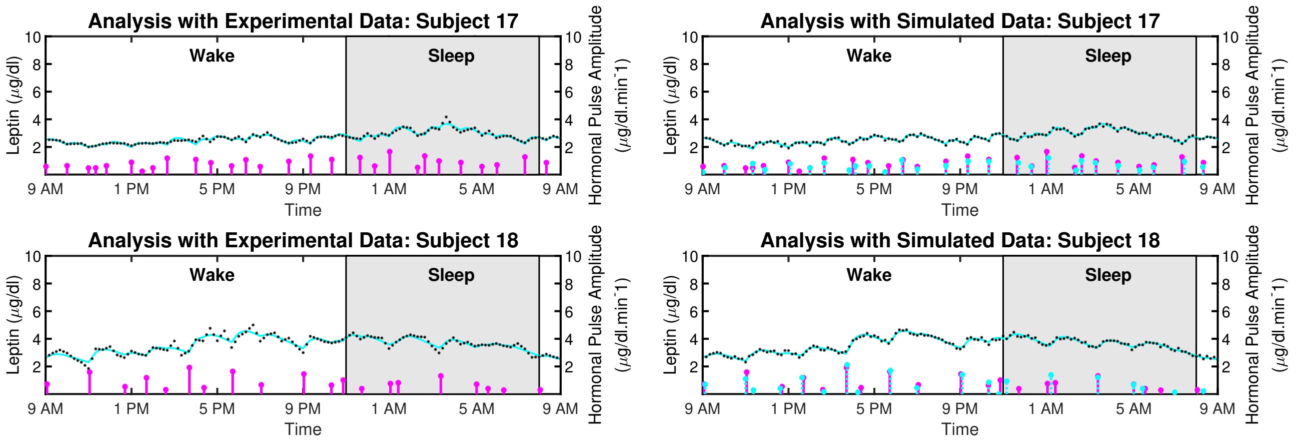

Figure 1. Deconvolved Twenty-Four Hours Leptin Levels in Women with Obesity. (a) Sub plot shows the measured 24-hour leptin time series (black diamonds), the reconstructed leptin levels (cyan curve), the estimated pulse timings and amplitudes (magenta vertical lines) for experimental data. (b) Sub plot shows the simulated 24-hour leptin time series (black diamonds), the reconstructed leptin levels (cyan curve), the simulated pulse timings and amplitudes (cyan vertical lines), the recovered pulse timings and amplitudes (magenta vertical lines) for simulated data.

1.3 Leptin-cortisol antagonism

The Pearson correlation coefficient is a measure of the strength of a linear association between two variables. Figure 3 shows the correlation plots for leptin and cortisol levels. We plot for both the measured and estimated levels to show the similarities. Except for in subjects 3, 10, 15, 17, and 18, we observed significant differences in the subjects (i.e., $p \leq 0.05$).

2 Supplementary Information: Methods

2.1 Leptin Model Formation

The system design for leptin is based on the previously reported state-space model for cortisol secretion and clearance process presented in[5]. The model provided by Faghih *et al.*[3], is based on the stochastic differential model. This model considers the first-order differential system of equations for leptin synthesis in the adipose tissue and clearance by the renal system. In this model, we intend to utilize the sparse nature of the hormonal secretory events along with other physiological constraints in a state-space model to estimate the amplitude and frequency of hormonal secretory events. The rate of change in leptin concentration in the adipose tissue is equal to the difference between leptin synthesis rate and the leptin infusion rate from adipose tissue into the blood. Similarly, the rate of change in leptin concentration in the plasma is equal to the difference between the leptin infusion rate from adipose tissue into the blood and the leptin clearance rate by the renal system[7]. The leptin secretion dynamics are represented as follows:

$$\frac{dx_1(t)}{dt} = -\gamma_1 x_1(t) + u_l(t) \qquad \text{(Adipose Tissue)} \tag{1}$$

$$\frac{dx_2(t)}{dt} = \gamma_1 x_1(t) - \gamma_2 x_2(t) \qquad \text{(Plasma)} \tag{2}$$

where $x_1(t)$ and $x_2(t)$ represent the effective leptin concentrations inside adipose tissue and leptin concentration in the blood plasma, respectively. The model parameters γ_1 and γ_2 represent the infusion rate of leptin by the adipose tissue and the clearance rate of leptin by the kidneys, respectively. Input $u_l(t)$ represents the abstraction of effective pulses mainly produced by the adipose tissue. $u_l(t)$ is modeled as a summation of delta functions with $u_l(t) = \sum_{i=1}^{N} q_i \delta(t-\tau_i)$ where q_i is the magnitude of the pulse initiated at time τ_i. If no pulse occurs at τ_i, q_i will equal zero. The pulses are assumed to occur at integer minutes, i.e., in a 24-hour period, there are 1440 distinct locations ($N = 1440$). The blood samples are collected every 10 minutes, for M samples ($M = 144$). Blood samples were collected beginning at y_0 and then assayed for leptin. Let $y_{t_{10}}, y_{t_{20}}, \ldots, y_{t_{10M}}$, $t_k : k = 10, 20, \ldots 10M$

$$y_{t_k} = x_2(t_k) + v_{t_k} \tag{3}$$

where y_{t_k} and v_{t_k} represent the observed leptin level and hormone measurement error at a time t_k, respectively. We model v_{t_k} random variables as i.i.d. Then, for the system output, we assume that the observed output value at time t_k will be equal to the

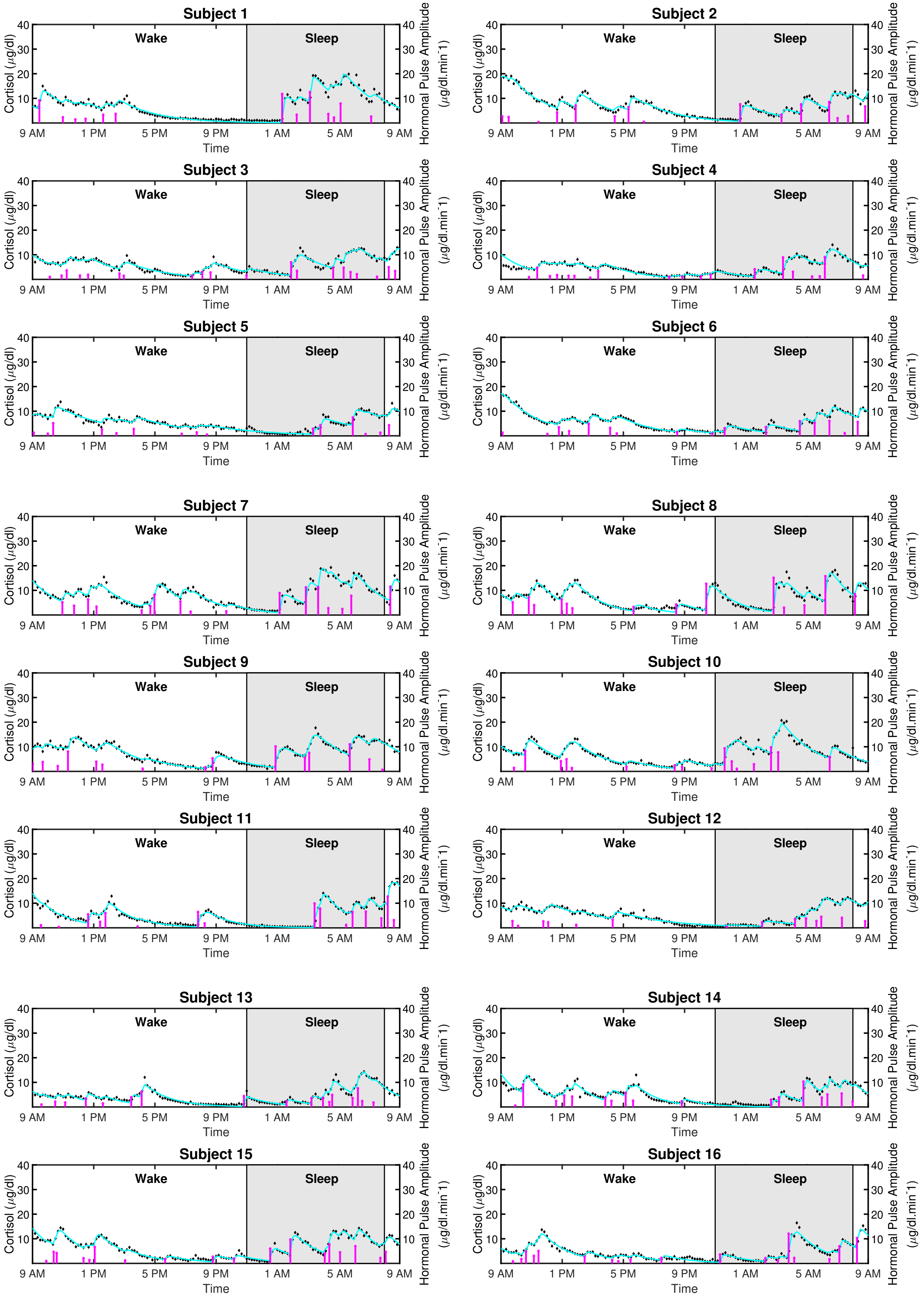

Subject 1
Subject 2
Subject 3
Subject 4
Subject 5
Subject 6
Subject 7
Subject 8
Subject 9
Subject 10
Subject 11
Subject 12
Subject 13
Subject 14
Subject 15
Subject 16
Wake
Sleep
Cortisol (μg/dl)
Hormonal Pulse Amplitude
(μg/dl.min^-1)
Time
9 AM
1 PM
5 PM
9 PM
1 AM
5 AM
9 AM
10
20
30
40

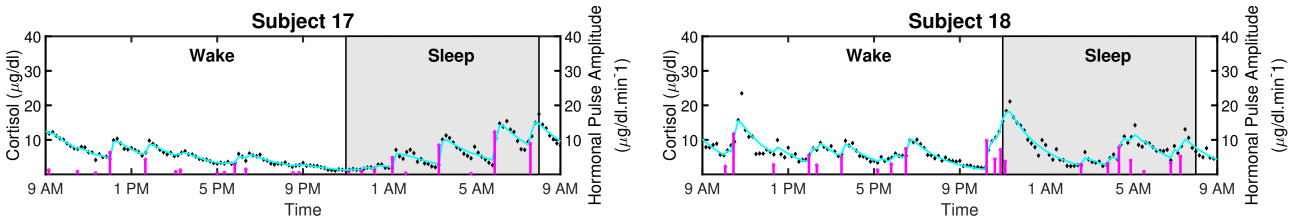

Figure 2. Deconvolved Experimental Twenty-Four Hours Cortisol Levels in Women with Obesity. Plot shows the measured 24-hour cortisol time series (black diamonds), the reconstructed cortisol levels (cyan curve), the estimated pulse timings and amplitudes (magenta vertical lines).

previous value, y_0, multiplied by a decay term and added to a secretion value and the error term. We can solve for the system over every t_k with the following equation:

$$\mathrm{y}_{l_{t_k}} = a_{t_k}\mathrm{y}_{l_0} + b_{t_k}\mathrm{u}_l + v_{t_k}$$

then, we solve for a_{t_k} and b_{t_k} using a forced solution approach, multiplying each side of the equation by e^{-at} and using mathematical methods to obtain the solutions.

$$\boldsymbol{b}_{t_k} = \left[\frac{\gamma_1}{\gamma_1-\gamma_2}(e^{-\gamma_2 k} - e^{-\gamma_1 k}) \quad \frac{\gamma_1}{\gamma_1-\gamma_2}(e^{-\gamma_2(k-1)} - e^{-\gamma_1(k-1)}) \quad \cdots \quad \frac{\gamma_1}{\gamma_1-\gamma_2}(e^{-\gamma_2} - e^{-\gamma_1}) \quad \underbrace{0 \quad \cdots \quad 0}_{N-k}\right]', \; a_{t_k} = e^{-\gamma_2 k}$$

and with u_l representing the input over the entire 24-hour sampling period, with values q_i over i=1,…,1440. From these matrices, we can form a combined representation for the system at any time, using:
$\mathbf{y}_l = \begin{bmatrix} y_{t_{10}} & y_{t_{20}} & \cdots & y_{t_{10M}} \end{bmatrix}'$, $\boldsymbol{\gamma} = \begin{bmatrix} \gamma_1 & \gamma_2 \end{bmatrix}'$, $\boldsymbol{A_\gamma} = \begin{bmatrix} a_{t_{10}} & a_{t_{20}} & \cdots & a_{t_{10M}} \end{bmatrix}'$, $\boldsymbol{B_\gamma} = \begin{bmatrix} b_{t_{10}} & b_{t_{20}} & \cdots & b_{t_{10M}} \end{bmatrix}'$, $\mathbf{u} = \begin{bmatrix} q_1 & q_2 & \cdots & q_N \end{bmatrix}'$, $\boldsymbol{v} = \begin{bmatrix} v_{t_{10}} & v_{t_{20}} & \cdots & v_{t_{10M}} \end{bmatrix}'$.
Hence, we represent the system output as:

$$\mathbf{y}_l = \boldsymbol{A_\gamma}\mathrm{y}_{l_0} + \boldsymbol{B_\gamma}\mathbf{u}_l + \boldsymbol{v}_l$$

where output vector $\mathbf{y}_l$ is dependent on the initial signal value, the input pulses, and the error term. The values of matrices $\boldsymbol{A_\gamma}$ and $\boldsymbol{B_\gamma}$ are dependent on the values of $\boldsymbol{\gamma}$ for the given subject.

2.2 Leptin Parameter Estimation

Sparse signal reconstruction allows us to recover information about the timing and amplitude of hormone pulses beginning with a discrete-time sampled signal. Following the data reported by[8], we develop a relationship between γ_1 and γ_2 assuming that the infusion rate of leptin into the adipose tissue is at least greater than or equal to the rate of leptin clearance from circulation by the kidneys. We use this relationship while estimating the model parameters (i.e., $\gamma_1 \geq \gamma_2$). The values of the infusion and clearance rates are assumed to be nonnegative (i.e., $\gamma_1, \gamma_2 \geq 0$).

Authors in[9] compared the leptin levels of women with obesity with healthy women. They found that the concentration of independent pulsatile parameters of leptin, such as pulse duration and frequency remained consistent between the groups. Moreover, they observed that the excessive leptin levels in persons with obesity are only due to an increase in pulse amplitude. The average number of leptin secretory events was found to be 32.0 ± 1.8 per day with 7-minute sampling[9], within a range of 29 to 39 pulses among the subjects. In a study done the following year, researchers in[10] found the average number of leptin pulses to be 30.0 ± 1 with the range of pulses between 21 to 39 over a 24-hour sampling period. These studies give a likely average of around 30 pulses per day for leptin, with a range between 20 to 40. Hence, we assume there to be between 20-40 secretory events during a 24-hour period, represented by the nonzero elements in our input function $u(t)$. Consequently, we formulate an optimization problem to generate values for u and γ that result in the minimum error for the output value. We consider the minimization of:

$$\frac{1}{2}||\mathbf{y}_l - \boldsymbol{A_\gamma}\mathrm{y}_{l_0} - \boldsymbol{B_\gamma}\mathbf{u}_l||_2^2$$

According to the previous section, it is equivalent to the error term $\boldsymbol{v}_l$. We also consider the corresponding constraints with the physiological boundary conditions (i.e., $20 \leq ||\mathbf{u}_l||_0 \leq 40$ and $\mathbf{u}_l \geq 0$).

We consider the amplitude of hormonal pulses to be greater than zero because these effective abstractions of pulses lead to the secretion of the hormone, and we cannot have negative pulses leading to the secretion of hormone.

Using the relationship between γ_1 and γ_2, we form the additional constraints as $C\gamma \leq b$ where $C = \begin{bmatrix} -1 & -1 & 0 \\ 1 & 0 & -1 \end{bmatrix}^\top$ and $b = \begin{bmatrix} 0 & 0 & 0 \end{bmatrix}^\top$. Each row of C and b are formed based on the physiological constraints on γ_1 and γ_2. We formulate the

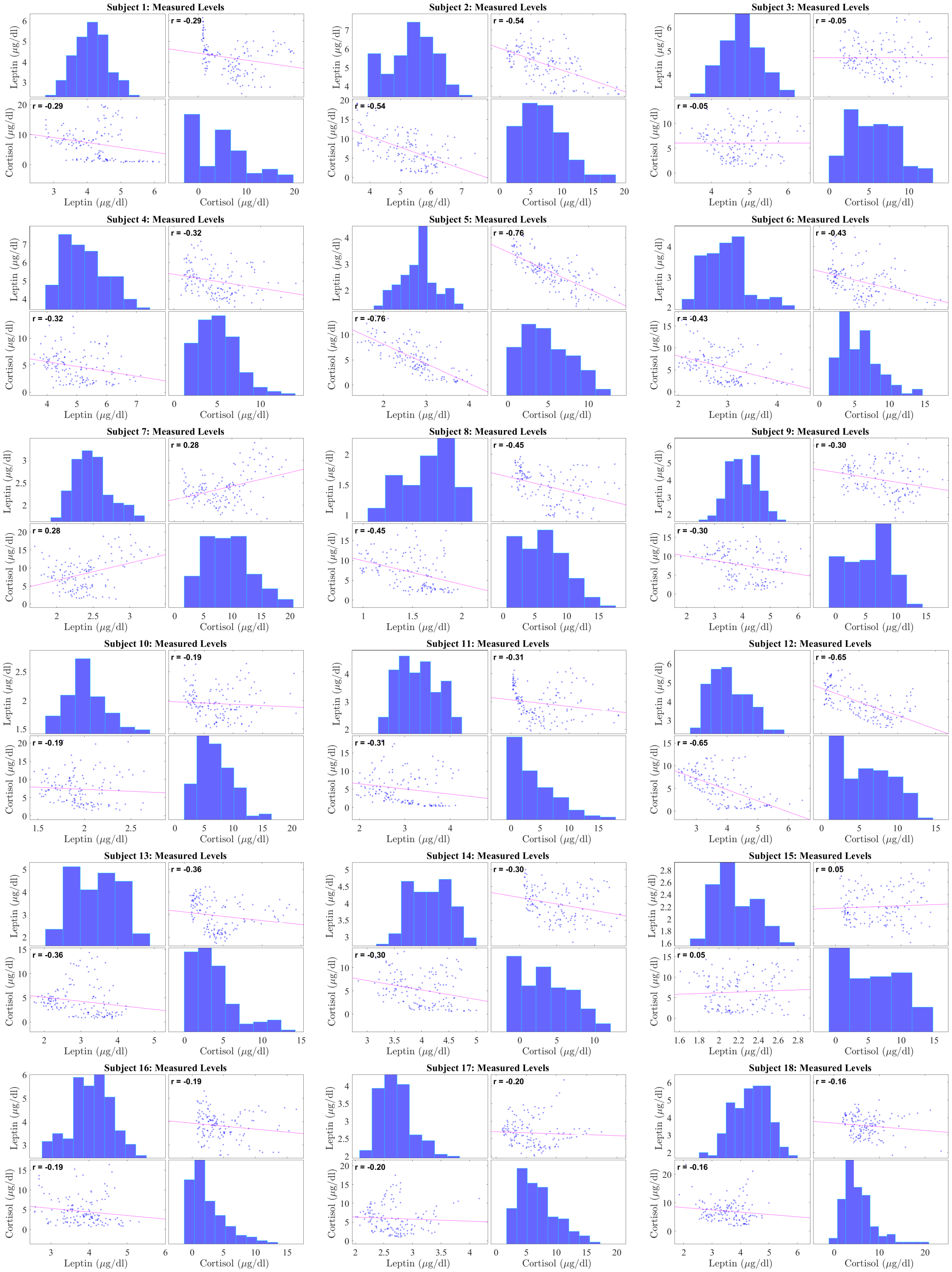
Subject 1: Measured Levels
r = -0.29
r = -0.29
Subject 2: Measured Levels
r = -0.54
r = -0.54
Subject 3: Measured Levels
r = -0.05
r = -0.05
Subject 4: Measured Levels
r = -0.32
r = -0.32
Subject 5: Measured Levels
r = -0.76
r = -0.76
Subject 6: Measured Levels
r = -0.43
r = -0.43
Subject 7: Measured Levels
r = 0.28
r = 0.28
Subject 8: Measured Levels
r = -0.45
r = -0.45
Subject 9: Measured Levels
r = -0.30
r = -0.30
Subject 10: Measured Levels
r = -0.19
r = -0.19
Subject 11: Measured Levels
r = -0.31
r = -0.31
Subject 12: Measured Levels
r = -0.65
r = -0.65
Subject 13: Measured Levels
r = -0.36
r = -0.36
Subject 14: Measured Levels
r = -0.30
r = -0.30
Subject 15: Measured Levels
r = 0.05
r = 0.05
Subject 16: Measured Levels
r = -0.19
r = -0.19
Subject 17: Measured Levels
r = -0.20
r = -0.20
Subject 18: Measured Levels
r = -0.16
r = -0.16
Leptin (μg/dl)
Cortisol (μg/dl)

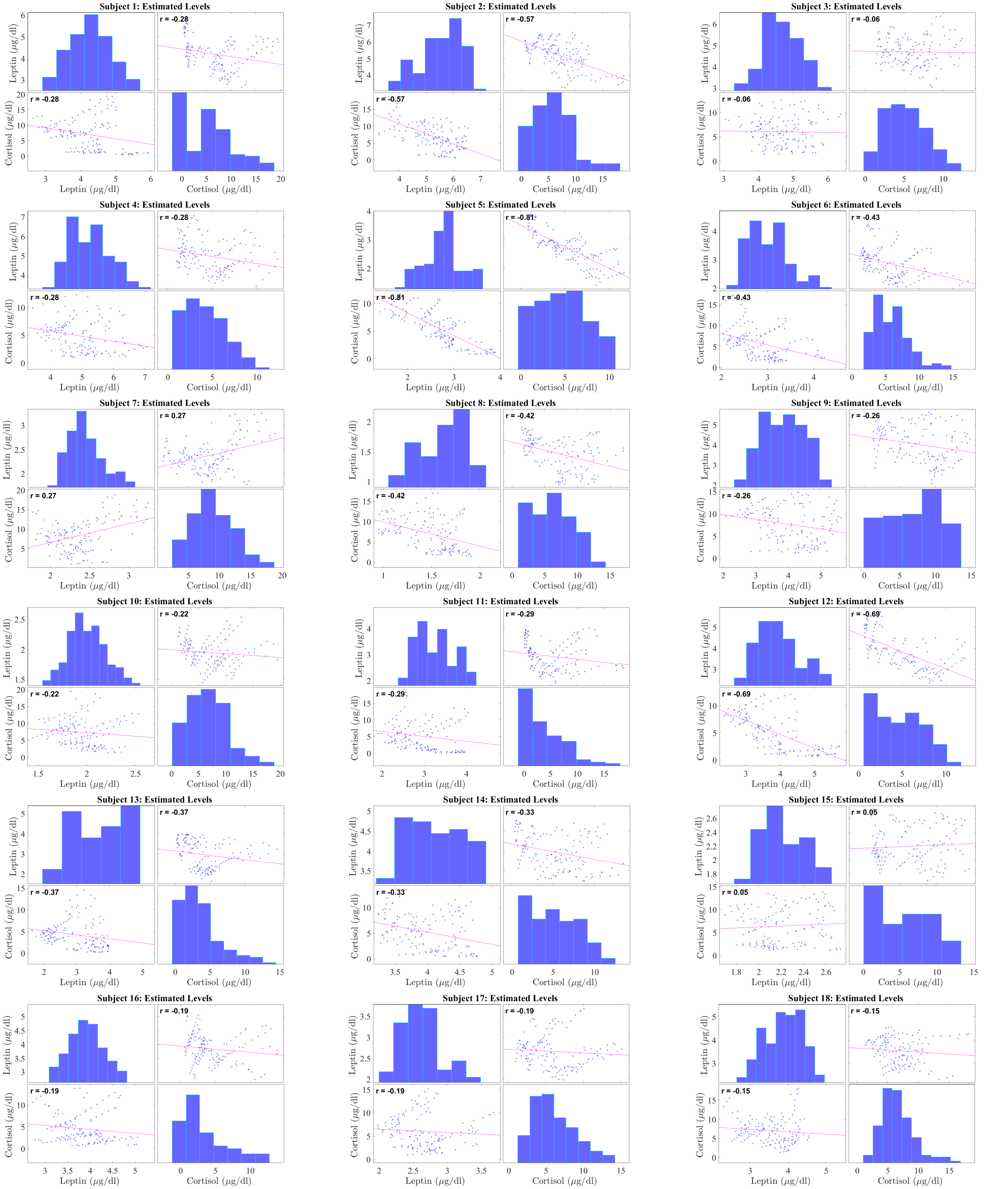

Figure 3. Correlation Plot between Leptin and Cortisol Levels in Women with Obesity. The correlation plots are as follows: The first eighteen plots left show the correlation between measured leptin and cortisol levels while the last eighteen plots show the correlation between estimated leptin and cortisol levels. Each correlation plot incorporates a histogram and a scatter plot: the top left plot shows a histogram depicting leptin distributions and the bottom right plot shows a histogram depicting cortisol distributions, the bottom left plot and top right plot shows the scatter plot depicting correlation between leptin and cortisol.

optimization problem as follows:

$$\min \frac{1}{2}||\mathbf{y}_l - \boldsymbol{A}_{\boldsymbol{\gamma}}\mathrm{y}_{l_0} - \boldsymbol{B}_{\boldsymbol{\gamma}}\mathbf{u}_l||_2^2$$
$$\text{s.t.}$$
$$20 \le ||\mathbf{u}_l||_0 \le 40$$
$$\mathbf{u}_l \ge 0$$
$$C\gamma \le b$$

where

$$C = \begin{bmatrix} -1 & -1 & 0 \\ 1 & 0 & -1 \end{bmatrix}^\top, b = \begin{bmatrix} 0 & 0 & 0 \end{bmatrix}^\top$$

The optimization problem is NP-hard. We consider the l_p-norm as an approximation to the l_0-norm, where p is between 0 and 2. We solve this problem by using an extension of the FOCal Under-determined System Solver (FOCUSS)[11] algorithm. The optimization problem is given as:

$$\min_{C\boldsymbol{\gamma}\le b} J_\lambda(\gamma, \mathbf{u}_l) = \frac{1}{2}||y - \boldsymbol{A}_\gamma \mathrm{y}_0 - B_\gamma \mathbf{u}_l||_2^2 + \lambda||\mathbf{u}_l||_p^p \tag{4}$$

Here, the value of λ is chosen to contain the number of pulses between 20 and 40. If λ is large, the minimization of the cost function will focus on the term it is contained in, and if λ is zero, we will have the original error minimization problem. This term balances the minimization of the error with returning the maximum sparsity for the input.

We solve this optimization problem iteratively by using a coordinate descent algorithm. We iterate between the following steps:

$$\mathbf{u}_l^{(m+1)} = \arg\min_{\boldsymbol{u}_l \ge 0} J_\lambda(\boldsymbol{\gamma}^{(m)}, \mathbf{u}_l) \tag{5}$$

$$\boldsymbol{\gamma}^{(m+1)} = \arg\min_{C\gamma\le \mathbf{b}} J_\lambda(\boldsymbol{\gamma}, \mathbf{u}_l^{(m+1)}). \tag{6}$$

2.3 Cortisol Model Formulation

In this research, we use the cortisol secretion model provided in[5]. Faghih *et al.*[5] exploits the sparse nature of hormonal secretory events and other physiological constraints to estimate the amplitude and timing of the secretory events using a state-space model. The rate at which cortisol concentration changes in the adrenal glands is equal to the difference between the rate at which it is infused in the blood and the rate at which it is secreted. The rate of change of plasma cortisol concentration is equal to the difference between the rate at which it is infused by the adrenal gland into the blood and the rate at which it is cleared by the liver from the blood:

$$\frac{dx_3(t)}{dt} = -\psi_1 x_3(t) + u_c(t) \qquad \text{(Adrenal Glands)} \tag{7}$$

$$\frac{dx_4(t)}{dt} = \psi_1 x_3(t) - \psi_2 x_4(t) \qquad \text{(Serum)} \tag{8}$$

where $x_3(t)$ and $x_4(t)$ represent the cortisol concentrations in the adrenal glands and the blood plasma, respectively. The physiological model parameters ψ_1 and ψ_2 represent the infusion rate of cortisol by the adrenal glands and the cortisol clearance rate by the liver. Input $u_c(t)$ represents the abstraction of pulses coming from the hypothalamus responsible for the production of cortisol. $u_c(t)$ can be modeled as a summation of delta functions with $u_c(t) = \sum_{i=1}^{N} q_i \delta(t - \tau_i)$. q_i is the magnitude of the pulse initiated at time τ_i. If no pulse occurs at τ_i, q_i will equal zero. The pulses are assumed to occur at integer minutes, i.e., in a 24-hour period, there are 1440 distinct locations ($N = 1440$). The blood samples are collected every 10 minutes for M samples ($M = 144$). As explained in Section 2.1, the measurement output can be represented as:

$$\mathrm{y}_{c_{t_k}} = x_4(t_k) + v_{t_k} \tag{9}$$

where y_{t_k} and v_{t_k} are the observed cortisol concentration and the measurement error, respectively. The initial concentration of cortisol in adrenal glands and plasma is assumed to be zero and y_{c_0}. The system is further expressed as:

$$\mathbf{y}_c = \boldsymbol{A}_{\boldsymbol{\psi}} \mathrm{y}_{c_0} + \boldsymbol{B}_{\boldsymbol{\psi}} \mathbf{u}_c + \boldsymbol{v}_c \tag{10}$$

where $\mathbf{y}_c = \begin{bmatrix} \mathrm{y}_{t_{10}} & \mathrm{y}_{t_{20}} & \cdots & \mathrm{y}_{t_{10M}} \end{bmatrix}'$, $\boldsymbol{\psi} = \begin{bmatrix} \psi_1 & \psi_2 \end{bmatrix}'$, $\boldsymbol{A}_{\boldsymbol{\psi}} = \begin{bmatrix} a_{t_{10}} & a_{t_{20}} & \cdots & a_{t_{10M}} \end{bmatrix}'$, $\boldsymbol{B}_{\boldsymbol{\psi}} = \begin{bmatrix} b_{t_{10}} & b_{t_{20}} & \cdots & b_{t_{10M}} \end{bmatrix}'$, $\mathbf{u} = \begin{bmatrix} q_1 & q_2 & \cdots & q_N \end{bmatrix}'$, $\boldsymbol{v}_c = \begin{bmatrix} v_{t_{10}} & v_{t_{20}} & \cdots & v_{t_{10M}} \end{bmatrix}'$, $a_{t_i} = e^{-\psi_2 i}$ and $\boldsymbol{b}_{t_i} = [\frac{\psi_1}{\psi_1 - \psi_2}(e^{-\psi_2 i} - e^{-\psi_1 i}) \quad \frac{\psi_1}{\psi_1 - \psi_2}(e^{-\psi_2 (i-1)} - e^{-\psi_1 (i-1)}) \quad \cdots \quad \frac{\psi_1}{\psi_1 - \psi_2}(e^{-\psi_2} - e^{-\psi_1}) \quad \underbrace{0 \ \cdots \ 0}_{N-i}]'$.

2.4 Cortisol Parameter Estimation

To estimate the model parameters, timings, and amplitudes of hormonal secretory events, we introduce some constraints in (10). We assume that the infusion rate of cortisol from the adrenal glands is at least four times the clearance rate of cortisol by the liver (i.e., $4\psi_2 \leq \psi_1$). In[53, 12], it has been shown that there exists between 15 to 22 cortisol secretory events (i.e., $15 \leq ||\mathbf{u}_c||_0 \leq 22, \mathbf{u}_c \geq 0$) in a 24-hour period. We therefore state the optimization problem as

$$\min_{\substack{\mathbf{u}_c \geq 0 \\ D\boldsymbol{\psi} \leq e}} J_\lambda(\psi, \mathbf{u}_c) = \frac{1}{2}||\mathbf{y}_c - \boldsymbol{A}_\psi y_{c_0} - \boldsymbol{B}_\psi \mathbf{u}_c||_2^2 + \lambda ||\mathbf{u}_c||_p^p \tag{11}$$

where $D = \begin{bmatrix} -1 & -1 & 0 \\ 4 & 0 & -1 \end{bmatrix}^\top$, $e = \begin{bmatrix} 0 & 0 & 0 \end{bmatrix}^\top$.

Similar to Section 2.2, the regularization parameter, i.e., λ, is selected such that the sparsity level of pulses, i.e. $||\mathbf{u}_c||_0$, remains within the physiologically plausible range. We choose the approximation for the $l_0 - \text{norm}$ as $l_p - \text{norm}$ $(0 < p \leq 2)$. This problem can be solved using a deconvolution algorithm, which uses the coordinate descent approach until we achieve convergence. We iterate between the following steps:

$$\mathbf{u}^{(n+1)} = \arg \min_{\boldsymbol{u}_c \geq 0} J_\lambda(\boldsymbol{\psi}^{(n)}, \mathbf{u}_c) \tag{12}$$

$$\boldsymbol{\psi}^{(n+1)} = \arg \min_{D\boldsymbol{\psi} \leq \mathbf{e}} J_\lambda(\boldsymbol{\psi}, \mathbf{u}_c^{(n+1)}). \tag{13}$$

2.5 Deconvolution

The same approach is used to solve the optimization problems for both leptin and cortisol but with different constraints. The FOCUSS algorithm can be used to solve the optimization problem in (5) and in (12)[11]. This algorithm uses a reweighted norm minimization approach. The FOCUSS algorithm uses the l_2-norm of the cost function to solve the minimization problem[11]. The deconvolution can be split into two stages with an initialization step using FOCUSS+, which is an extension of the FOCUSS algorithm, followed by a coordinate descent approach using Generalized Cross-Validation (GCV) - FOCUSS[13]. Coordinate descent approach minimizes multi-variable along one coordinate direction at a time[14]. For the cost function J_λ, our problem must be minimized in two directions: the model parameters and u directions. For leptin, let $\zeta_1 = \gamma_1$, $\zeta_2 = \gamma_2$ and $\mathbf{u}_l = \mathbf{u}$. Similarly for cortisol, let $\zeta_1 = \psi_1$, $\zeta_2 = \psi_2$ and $\mathbf{u}_c = \mathbf{u}$

2.5.1 Initialization

We choose the initial estimates for $\mathbf{u}$ and $\boldsymbol{\zeta}$ to be zero and the solution is found by refining this estimate for each iteration until the localized solution is derived. The initialization algorithm uses the interior point method to solve for the values of ζ once a solution is estimated for $\mathbf{u}$ using the FOCUSS+ algorithm. We iterate between the optimization problems to get a proper estimate.

FOCUSS+[15] solves for nonnegative solutions while maintaining the sparsity of $\mathbf{u}$. λ is updated using an heuristic approach. We maintain the trade-off between residual error and sparsity by updating λ iteratively until it reaches maximum regularization $\lambda_{\max}$. The residual error $||\mathbf{y}_{\boldsymbol{\zeta}} - \boldsymbol{B}_{\boldsymbol{\zeta}}\mathbf{u}||$ reduces with every iteration. It allows us to constrain the maximum number of non-zero elements in $\mathbf{u}$ at r (where, in healthy individuals, r is 22 for cortisol and 40 for leptin). We relax these constraints since the subjects suffer from obesity (r is 25 for cortisol and 45 for leptin).

FOCUSS+ works as follows:

Algorithm 1 FOCUSS+

1: $F_{\mathbf{u}}=\text{diag}(|\mathbf{u}_i^{(s)}|^{2-p})$
2: $\lambda^{(s)} = \left(1 - \frac{||\mathbf{y}_\zeta - \boldsymbol{B}_\zeta \mathbf{u}||_2}{||\mathbf{y}_\zeta||_2}\right)\lambda_{\mathbf{max}}, \lambda > 0$
3: $\mathbf{u}^{(s+1)} = F_{\mathbf{u}}^{(s)} \boldsymbol{B}_\zeta^T (B_\zeta F_{\mathbf{u}}^{(s)} \boldsymbol{B}_\zeta^T + \lambda^{(l)}\mathbf{I})^{-1} y_\phi$
4: $\mathbf{u}_i^{(s+1)} \leq 0 \rightarrow \mathbf{u}_i^{(s+1)} = 0$
5: After more than 50% iterations, if $||\mathbf{u}^{(s+1)}||_0 > r$, select the largest n elements of $\mathbf{u}^{(s+1)}$and set the rest to zero.
6: Iterate until convergence.

The initialization algorithm is given as:

Algorithm 2 Initialization

1: Initialize $\bar{\boldsymbol{\zeta}}^{\mathbf{0}}$ by sampling a uniform random variable w in [0,1] and let $\bar{\boldsymbol{\zeta}}^{\mathbf{0}}$=[$w$ $\frac{w}{4}$].
2: **for** `j= 1,2,3,...,30` **do**
3: Set $\bar{\boldsymbol{\zeta}}$ equal to $\bar{\boldsymbol{\zeta}}^{j-1}$; using FOCUSS+[15], solve for $\bar{\boldsymbol{u}}^{\boldsymbol{j}}$ by initializing the optimization problem in (5) and (12) at a vector of all ones
4: Set $\bar{\boldsymbol{u}}$ equal to $\bar{\boldsymbol{u}}^{j}$; using the interior point method, solve for $\bar{\boldsymbol{\zeta}}^{\boldsymbol{j}}$ by initializing the optimization problem in (6) and (13) at $\bar{\boldsymbol{\zeta}}^{j-1}$
5: **end for**

2.5.2 Co-ordinate Descent Approach

FOCUSS+ usually takes about 10 to 50 iterations to converge[15]. Though we obtain an estimate for $\boldsymbol{\zeta}$ and $\mathbf{u}$ by iteratively solving for it, we also need to find a good estimate for λ to maintain a balanced trade-off between λ and the sparsity of $\mathbf{u}$. The GCV technique is used to obtain an estimate for the regularization parameter[13]. GCV function is given as follows:

$$G(\lambda) = \frac{L||(1-B_\zeta F_{\mathbf{u}}^{(s)} B_\zeta^T (B_\zeta F_{\mathbf{u}}^{(s)} B_\zeta^T + \lambda^{(s)}\mathbf{I})^{-1}) y_\zeta||^2}{(\text{trace}(1-B_\zeta F_{\mathbf{u}}^{(s)} B_\zeta^T (B_\zeta F_{\mathbf{u}}^{(s)} B_\zeta^T + \lambda^{(s)} I)^{-1}))^2}$$

L is the number of data points. s is the estimated value at s^{th} iteration of GCV-FOCUSS+ algorithm. The use of the GCV technique and FOCUSS+ together allows us to find a reasonable choice for λ and further helps us to filter out noise to estimate $\mathbf{u}$[5, 16]. GCV-FOCUSS+ works as follows:

Algorithm 3 GCV-FOCUSS+

1: Initialize j=0 (Iteration Counter)
2: $F_u^{(j)} = \text{diag}(|u_i^{(j)}|^{2-p}$
3: $\mathbf{u}^{(j+1)} = F_{\mathbf{u}}^{(j)} B_\phi^T (B_\phi F_{\mathbf{u}}^{(j)} B_\phi^T + \lambda^{(j)} I)^{-1} y_\phi$
4: $\mathbf{u}_i^{(j+1)} \leq 0 \rightarrow \mathbf{u}_i^{(j+1)} = 0$
5:
$$\lambda^{(j+1)} = \arg \min_{0 \leq \lambda \leq 10} G(\lambda)$$
6: Iterate until convergence

The deconvolution algorithm works as follows:

Algorithm 4 Deconvolution Algorithm

1: Initialize $\bar{\boldsymbol{u}}^{\mathbf{0}}$ and $\bar{\boldsymbol{\zeta}}^{\mathbf{0}}$ by setting them equal to $\bar{\boldsymbol{u}}^{\boldsymbol{s}}$ and $\bar{\boldsymbol{\zeta}}^{\boldsymbol{s}}$ obtained from Algorithm 2.
2: **while** until convergence **do**
3: Set $\bar{\boldsymbol{\zeta}}$ equal to $\bar{\boldsymbol{\zeta}}^{l-1}$; using GCV-FOCUSS+[5], solve for $\bar{\boldsymbol{u}}^{\boldsymbol{l}}$ by initializing the optimization problem (5)/(12) at $\bar{\boldsymbol{u}}^{l-1}$
4: Set $\bar{\boldsymbol{u}}$ equal to $\bar{\boldsymbol{u}}^{l}$; using the interior point method, solve for $\bar{\boldsymbol{\zeta}}^{\boldsymbol{l}}$ by initializing the optimization problem in (6)/(13) at $\bar{\boldsymbol{\zeta}}^{l-1}$
5: **end while**
6: Repeat the above steps for several initializations and set the estimated model parameter $\overline{\phi}$ and input $\overline{u}$ equal to values that minimize J_λ in (4)/(11).

References

1. Simon, C., Gronfier, C., Schlienger, J. & Brandenberger, G. Circadian and ultradian variations of leptin in normal man under continuous enteral nutrition: relationship to sleep and body temperature. *The J. Clin. Endocrinol. & Metab.* **83**, 1893–1899 (1998).

2. Aschbacher, K. *et al.* The hypothalamic–pituitary–adrenal–leptin axis and metabolic health: a systems approach to resilience, robustness and control. *Interface focus* **4**, 20140020 (2014).

3. Brown, E. N., Meehan, P. M. & Dempster, A. P. A stochastic differential equation model of diurnal cortisol patterns. *Am. J. Physiol. And Metab.* **280**, E450–E461 (2001).

4. Pednekar, D. D., Amin, M. R., Fekri, H., Aschbacher, C. L. J., Kirstin & Faghih, R. T. A system theoretic investigation of cortisol dysregulation in fibromyalgia patients with chronic fatigue. In *Engineering in Medicine and Biology Society (EMBC), 2019 41st Annual International Conference of the IEEE* (IEEE, 2019).

5. Faghih, R. T., Dahleh, M. A., Adler, G. K., Klerman, E. B. & Brown, E. N. Deconvolution of serum cortisol levels by using compressed sensing. *PloS one* **9**, e85204 (2014).

6. Faghih, R. T., Dahleh, M. A., Adler, G. K., Klerman, E. B. & Brown, E. N. Quantifying pituitary-adrenal dynamics and deconvolution of concurrent cortisol and adrenocorticotropic hormone data by compressed sensing. *IEEE Transactions on Biomed. Eng.* **62**, 2379–2388 (2015).

7. Wolf, G., Chen, S., Han, D. C. & Ziyadeh, F. N. Leptin and renal disease. *Am. J. Kidney Dis.* **39**, 1–11 (2002).

8. Klein, S., Coppack, S. W., Mohamed-Ali, V. & Landt, M. Adipose tissue leptin production and plasma leptin kinetics in humans. *Diabetes* **45**, 984–987 (1996).

9. Licinio, J. *et al.* Human leptin levels are pulsatile and inversely related to pituitary–ardenal function. *Nat. medicine* **3**, 575 (1997).

10. Licinio, J. *et al.* Sex differences in circulating human leptin pulse amplitude: clinical implications. *The J. Clin. Endocrinol. & Metab.* **83**, 4140–4147 (1998).

11. Gorodnitsky, I. F. & Rao, B. D. Sparse signal reconstruction from limited data using focuss: A re-weighted minimum norm algorithm. *IEEE Transactions on signal processing* **45**, 600–616 (1997).

12. Veldhuis, J. D., Iranmanesh, A., Lizarralde, G. & Johnson, M. L. Amplitude modulation of a burstlike mode of cortisol secretion subserves the circadian glucocorticoid rhythm. *Am. J. Physiol. And Metab.* **257**, E6–E14 (1989).

13. Golub, G. H., Heath, M. & Wahba, G. Generalized cross-validation as a method for choosing a good ridge parameter. *Technometrics* **21**, 215–223 (1979).

14. Wright, S. J. Coordinate descent algorithms. *Math. Program.* **151**, 3–34 (2015).

15. Murray, J. F. *Visual recognition, inference and coding using learned sparse overcomplete representations*. Ph.D. thesis, University of California, San Diego (2005).

16. Zdunek, R. & Cichocki, A. Improved m-focuss algorithm with overlapping blocks for locally smooth sparse signals. *IEEE Transactions on Signal Process.* **56**, 4752–4761 (2008).
